# Bone and soft tissue sarcoma mortality in 19 811 patients diagnosed in Japan, 2006-2020

**DOI:** 10.1093/jncics/pkag001

**Published:** 2026-01-07

**Authors:** Kengo Kawaguchi, Makoto Endo, Haruhisa Fukuda, Akira Kawai, Toshifumi Fujiwara, Akira Nabeshima, Nobuhiko Yokoyama, Yoshinao Oda, Yasuharu Nakashima

**Affiliations:** Department of Orthopaedic Surgery, Graduate School of Medical Sciences, Kyushu University, Fukuoka, Japan; Department of Health Care Administration and Management, Graduate School of Medical Sciences, Kyushu University, Fukuoka, Japan; Department of Orthopaedic Surgery, Graduate School of Medical Sciences, Kyushu University, Fukuoka, Japan; Department of Health Care Administration and Management, Graduate School of Medical Sciences, Kyushu University, Fukuoka, Japan; Division of Musculoskeletal Oncology and Rehabilitation, National Cancer Center Hospital, Tokyo, Japan; Department of Orthopaedic Surgery, Graduate School of Medical Sciences, Kyushu University, Fukuoka, Japan; Department of Orthopaedic Surgery, Graduate School of Medical Sciences, Kyushu University, Fukuoka, Japan; Department of Orthopaedic Surgery, Graduate School of Medical Sciences, Kyushu University, Fukuoka, Japan; Department of Anatomic Pathology, Graduate School of Medical Sciences, Kyushu University, Fukuoka, Japan; Department of Orthopaedic Surgery, Graduate School of Medical Sciences, Kyushu University, Fukuoka, Japan

## Abstract

**Background:**

Sarcomas are rare malignant tumors with heterogeneous histologies and limited population-based evidence. Although new treatments have been introduced in recent years, their effect on real-world survival outcomes remains unclear. This study aimed to evaluate recent trends in mortality for bone and soft tissue sarcomas in Japan.

**Methods:**

We conducted a cohort study using data from the Bone and Soft Tissue Tumor Registry, a nationwide database maintained by the Japanese Orthopaedic Association. Patients diagnosed with primary sarcomas between 2006 and 2020 were included and grouped by diagnostic period (2006-2010, 2011-2015, 2016-2020). The primary outcome was cumulative mortality risk, estimated using Poisson regression. Subgroup analyses were conducted by age, sex, tumor origin, metastasis status, treatment modality, and histological subtype. Sensitivity analyses included multiple imputation, Kaplan–Meier estimates, and competing risk models.

**Results:**

A total of 19 811 patients were analyzed. No statistically significant change in overall mortality risk was observed across diagnostic periods. Ewing sarcoma showed a consistent decline in mortality, whereas other subtypes did not. Mortality risk was lower in patients who received surgery and higher in those who received radiotherapy or chemotherapy. Results were robust across sensitivity analyses.

**Conclusions:**

Survival outcomes for sarcoma patients in Japan have remained largely unchanged over the past 15 years, except for Ewing sarcoma. Novel therapeutic approaches are needed to achieve meaningful improvements in prognosis.

## Introduction

Sarcomas are malignant tumors originating from mesenchymal cells that primarily occur in bones and soft tissues.^1^ Compared with carcinomas, which arise from epithelial cells, sarcomas are rare, and many of the subtypes fall into the category of rare cancers. Consequently, sarcoma research experiences slow progress, leaving many aspects unexplored. Sarcomas are more common in younger individuals, making their treatment a important social priority.^2^

In recent years, several new treatments have been approved for bone and soft tissue sarcomas. In Japan, pazopanib, trabectedin, and eribulin were approved in 2012, 2015, and 2016, respectively, for the treatment of soft tissue sarcomas. Advances in chemotherapy-based treatments are expected to gradually improve the prognosis of patients with sarcomas. However, most studies on the temporal trends in the prognosis of bone and soft tissue sarcomas are outdated. Periodic reports exist on osteosarcoma; the most recent report included patients diagnosed only up to 2017.^3-5^ For other primary bone and soft tissue sarcomas, the latest studies covered only patients diagnosed up to 2014.^6-8^ Moreover, most of these studies focused on comparisons with decades-old data and did not reflect recent trends. Additionally, the sample sizes in these studies were limited, with fewer than 5000 participants, and were often restricted by age, clinical stage, and subtype. In a clinical setting, epidemiological data on the target disease are essential for evaluating treatments and informing patients. However, comprehensive descriptive epidemiological studies on bone and soft tissue sarcomas are scarce. Consequently, physicians must rely on small-scale studies, clinical trial results that may not reflect real-world data, or their own clinical experiences when making treatment decisions. Updating the knowledge of prognostic trends in bone and soft tissue sarcomas is therefore crucial.

The aim of this study was to clarify trends in mortality for bone and soft tissue sarcomas over nearly 15 years and to provide valuable information for clinical practice.

## Methods

### Study design

This cohort study used data from the Bone and Soft Tissue Tumor Registry in Japan for patients diagnosed between 2006 and 2020. Patients with newly diagnosed sarcomas were classified into 3 groups based on the period of diagnosis: 2006-2010, 2011-2015, and 2016-2020. Cumulative mortality risk was compared between the groups.

This study was conducted in accordance with the principles of the Declaration of Helsinki and was approved by the Bone and Soft Tissue Tumor Committee of the Japanese Orthopaedic Association and the Kyushu University ethics review board (Approval No. 24228). The need for written informed consent was waived by the institutional review board, as the study was conducted using an opt-out approach.

### Study setting

The Bone and Soft Tissue Tumor Registry in Japan is a nationwide organ-specific cancer registry operated by the Japanese Orthopaedic Association. Established in the 1950s, standardized electronic data have been collected since 2006. Diagnoses in this registry were confirmed at each institution; however, a central pathological review was not conducted. Further details are available in previous reports.^9^^,^^10^ We extracted data, including follow-up information up to December 2023, from the registry. Data collection was conducted in January 2025, and analyses were performed between February and March 2025.

### Study cohort

Patients who were diagnosed with malignant tumors since 2006 and registered in the Bone and Soft Tissue Tumor Registry were included in this study. The following were excluded: (1) nonprimary patients, including those who received treatment at another institution before registration; (2) patients without a pathological diagnosis because pathology is essential for diagnosing sarcomas; (3) nonsarcoma patients including those with carcinomas and lymphomas and those with special subtypes, such as gastrointestinal stromal tumors, intimal sarcomas, Kaposi sarcomas, and tumors with extremely rare malignant forms (eg, perineurioma and schwannoma), or patients with unclear diagnoses. Because this registry is led by the Japanese Orthopaedic Association and primarily captures tumors treated in orthopedic departments, sarcomas arising in organ systems mainly managed by other specialties, such as gynecologic or gastrointestinal sarcomas, were rarely registered. To avoid substantial selection bias related to these underrepresented sites, these tumors were not included in the present analyses. For patients diagnosed based on the previous World Health Organization classification criteria, pathology annotations were reviewed, and reclassification was performed according to the latest World Health Organization classification.^11^

### Outcome and other variables

The primary outcome was all-cause mortality, defined as death from disease and death from other causes, based on prognostic information recorded in the database. As a secondary endpoint, we examined temporal changes in the proportion of patients who underwent amputation.

The patients were categorized into 4 age groups: pediatric (younger than 15 years), adolescent and young adult (15-39 years), adult (40-64 years), and older adult (65 years and older). Sex was defined based on the biological sex. The origin type was classified as bone or soft tissue based on the tumor site. The patients were considered to have undergone surgery, radiotherapy, or chemotherapy based on whether the treatment was administered during the observation period, without considering the timing of administration. The definitions of translocation-related sarcomas and sarcomas with complex genomics are provided in Table S1 and Table S2, respectively.

### Statistical analysis

Following a previous study,^12^ we standardized the follow-up period across diagnostic periods by setting specific cutoffs of 10 years for the 2006-2010 cohort, 7 years for the 2011-2015 cohort, and 3 years for the 2016-2020 cohort. Poisson regression was used to estimate the number of deaths every 6 months, with the at-risk population in each period set as an offset. Based on the estimated mortality rates, the cumulative mortality risk was calculated as 1-exp (−cumulative rate). To smoothen the estimates and visualize the trends, a natural spline function (degrees = 3, *df* = 3) was applied. Subgroup analyses based on the clinical factors were performed using the same methods. All the analyses were performed as complete case studies.

We performed 3 sensitivity analyses. First, missing values were imputed using multiple imputations with the predictive mean matching method with 5 imputations, and Poisson regression was applied in the same manner as in the main analysis to compare cumulative mortality risk. Second, the Kaplan–Meier method was used to compare cumulative mortality. Third, competing risk analysis was performed using the cumulative incidence function, with death from other causes as a competing risk factor for death from disease. For Kaplan–Meier and competing risk analyses, complete case analyses were applied, and no follow-up period cutoff was set; instead, the entire follow-up period was evaluated.

For the secondary endpoint, temporal changes in amputation were evaluated among patients with limb primaries. We calculated the number and proportion of patients who underwent amputation in each diagnosis period (2006-2010, 2011-2015, and 2016-2020). To adjust for differences in follow-up duration, we performed logistic regression analysis with amputation status at the last follow-up as the outcome variable, using diagnosis period as the main explanatory variable and observation time (months) as a covariate. This analysis was performed as a complete case analysis, excluding patients with missing data in the variables of interest.

For all analyses, 95% confidence intervals (CIs) were calculated and are presented in the figures. Data preprocessing, statistical analyses, and visualization were performed using *R* version 4.4.2 (R Foundation for Statistical Computing, Vienna, Austria).

## Results

### Patient characteristics

**Figure 1.**
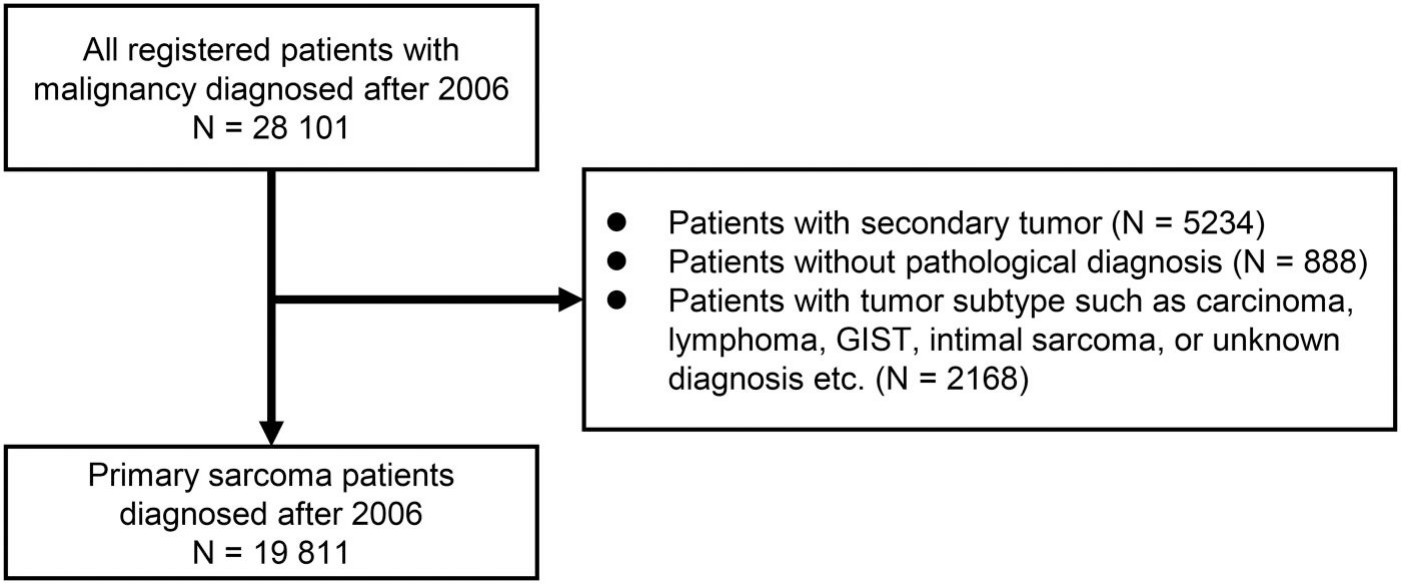
Flowchart of study participant selection. Abbreviation: GIST = gastrointestinal stromal tumor.

After applying the exclusion criteria to 28 101 participants extracted from the database, 19 811 patients with primary bone and soft tissue sarcomas were included in the final analysis (Figure 1). Table 1 summarizes the clinical characteristics of each diagnostic period group. The patients in the more recent diagnostic period tended to be older. Men accounted for a slightly higher proportion of patients than women. Approximately 10% of the patients had metastases at initial diagnosis, with a slight decrease in more recent diagnostic period groups. Among the histological subtypes, well-differentiated liposarcoma (also termed atypical lipomatous tumor) was the most common, followed by undifferentiated pleomorphic sarcoma and myxofibrosarcoma. Regarding treatment, 70%-80% of patients underwent surgery, approximately 20% received radiotherapy, and approximately 30% received chemotherapy.

**Table 1.** Clinical characteristics of patients by diagnostic period^a^

Variables	Groups		
	2006-2010	2011-2015	2016-2020
	*n* = 4771	*n* = 6814	*n* = 8226
Age at diagnosis, median (IQR), y	57 (37-70)	62 (43-72)	63 (46-74)
Age category, No. (%)			
Pediatric, younger than 15 y	301 (6.3)	335 (4.9)	325 (4.0)
Pediatric, younger than 15 y	1040 (21.8)	1150 (16.9)	1172 (14.2)
Adult, 40-64 y	1693 (35.5)	2392 (35.1)	2819 (34.3)
Older adult, 65 y and older	1737 (36.4)	2937 (43.1)	3910 (47.5)
Sex, No. (%)			
Women	2138 (44.8)	2959 (43.4)	3503 (42.6)
Origin type, No. (%)			
Bone	1464 (30.7)	1714 (25.2)	1835 (22.3)
Site, No. (%)			
Head and neck	85 (1.8)	145 (2.1)	197 (2.4)
Upper limb	515 (10.8)	744 (10.9)	918 (11.2)
Lower limb	2623 (55.0)	3566 (52.3)	4036 (49.1)
Trunk	1448 (30.4)	2229 (32.7)	2936 (35.7)
Size, median (IQR)	9.0 (6.0-13.0)	9.0 (6.0-13.0)	8.9 (5.7-13.0)
Metastasis at diagnosis, No. (%)			
Yes	547 (11.5)	708 (10.4)	798 (9.7)
Biopsy, No. (%)			
Yes	4007 (84.0)	5755 (84.5)	7098 (86.3)
Diagnosis, No. (%)			
Well-differentiated liposarcoma (atypical lipomatous tumor)	737 (15.4)	1211 (17.8)	1459 (17.7)
Undifferentiated pleomorphic sarcoma	676 (14.2)	998 (14.6)	1147 (13.9)
Myxofibrosarcoma	281 (5.9)	580 (8.5)	898 (10.9)
Conventional osteosarcoma	621 (13.0)	644 (9.5)	661 (8.0)
Dedifferentiated liposarcoma	100 (2.1)	305 (4.5)	578 (7.0)
Chondrosarcoma	368 (7.7)	459 (6.7)	519 (6.3)
Myxoid liposarcoma	384 (8.0)	460 (6.8)	490 (6.0)
Leiomyosarcoma	217 (4.5)	348 (5.1)	398 (4.8)
Synovial sarcoma	190 (4.0)	227 (3.3)	240 (2.9)
Malignant peripheral nerve sheath tumor	154 (3.2)	233 (3.4)	233 (2.8)
Ewing sarcoma	175 (3.7)	193 (2.8)	179 (2.2)
Chordoma	102 (2.1)	134 (2.0)	140 (1.7)
Pleomorphic liposarcoma	69 (1.4)	78 (1.1)	120 (1.5)
Others^b^	697 (14.6)	944 (13.9)	1164 (14.2)
Operation, No. (%)			
Yes	3818 (80.0)	5262 (77.2)	6180 (75.1)
Radiotherapy, No. (%)			
Yes	1139 (23.9)	1445 (21.2)	1830 (22.2)
Chemotherapy, No. (%)			
Yes	1673 (35.1)	2165 (31.8)	2516 (30.6)
Overall survival, median (IQR), mo	49.0 (20.0-90.0)	43.0 (18.0-70.0)	31.0 (16.0-41.0)
Prognosis, recurrence, No. (%)			
Yes	520 (10.9)	725 (10.6)	770 (9.4)
Not resected	454 (9.5)	767 (11.3)	1028 (12.5)
Prognosis, metastasis, No. (%)			
Yes	1493 (31.3)	1986 (29.1)	2264 (27.5)
Last follow-up status, No. (%)			
Died of disease	1100 (23.1)	1424 (20.9)	1383 (16.8)
Died of other causes	150 (3.1)	179 (2.6)	154 (1.9)
Alive with disease	568 (11.9)	1040 (15.3)	1560 (19.0)
No evidence of disease	2953 (61.9)	4171 (61.2)	5129 (62.4)

Abbreviation: IQR = interquartile range.

a Missing data is not shown.

b Only subtypes with *n* greater than 100 in the 2016-2020 group are shown; others are grouped as “Others.”

### Trends in cumulative mortality risk: overall and by clinical factors

**Figure 2.**
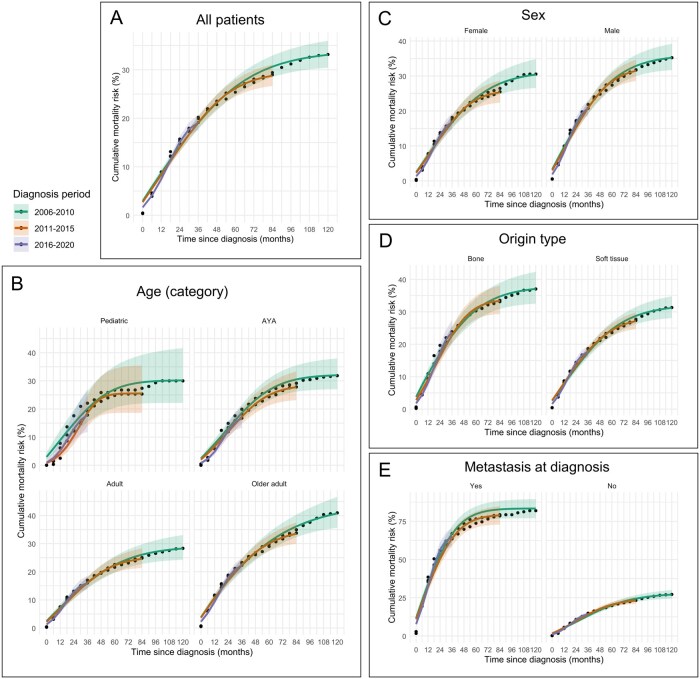
Trends in cumulative mortality risk by overall and clinical factors. Cumulative mortality risk for patients diagnosed during 2006-2010 (**green**), 2011-2015 (**orange**), and 2016-2020 (**purple**). The **shaded areas** represent 95% confidence intervals. Overall, the cumulative mortality risk was similar across the diagnostic periods **(A)**. Pediatric patients showed a tendency toward a lower cumulative mortality risk after 2011 than during 2006-2010, but the difference was not significant **(B)**. No statistically significant differences in cumulative mortality risk were observed according to sex **(C)**, tumor origin **(D)**, or the presence of metastasis at initial diagnosis **(E)** across the diagnostic periods. Abbreviation: AYA = adolescent and young adult.

Overall, the cumulative mortality risk did not decrease across the diagnostic period, indicating that survival outcomes have not improved (Figure 2, A). Pediatric patients had a consistently lower mortality risk after 2011 than during 2006-2010, but the difference was not statistically significant (Figure 2, B). Similarly, no clear differences were observed based on sex (Figure 2, C), the tumor origin (bone vs soft tissue) (Figure 2, D), or presence of distant metastases at initial diagnosis (Figure 2, E).

### Trends in cumulative mortality risk by treatment modality

**Figure 3.**
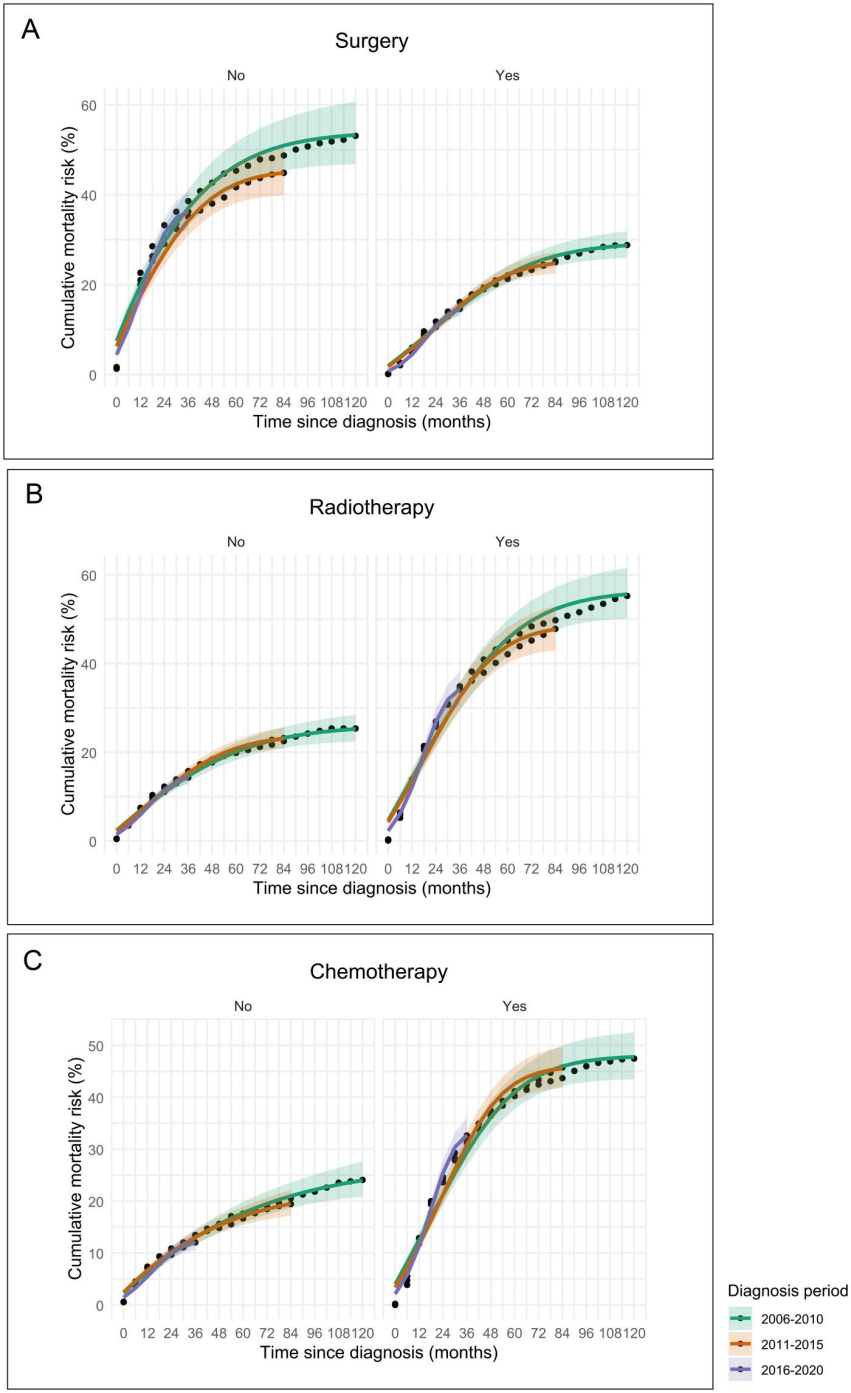
Trends in cumulative mortality risk by treatment modality. Cumulative mortality risk by treatment modality: surgery **(A)**, radiotherapy **(B)**, and chemotherapy **(C)**. No consistent changes in mortality trends were observed across diagnostic periods. However, patients who did not undergo surgery **(A)**, received radiotherapy **(B)**, or received chemotherapy **(C)** had a higher cumulative mortality risk than their respective counterparts.

When comparing the cumulative mortality risk based on treatment modality, patients who underwent surgery, those who did not receive radiotherapy, or those who did not receive chemotherapy had statistically significantly lower cumulative mortality risks than their respective counterparts (Figure 3). However, no consistent changes in the cumulative mortality risk were observed across the diagnostic periods for any treatment modality.

### Trends in cumulative mortality risk based on diagnostic subtype

**Figure 4.**
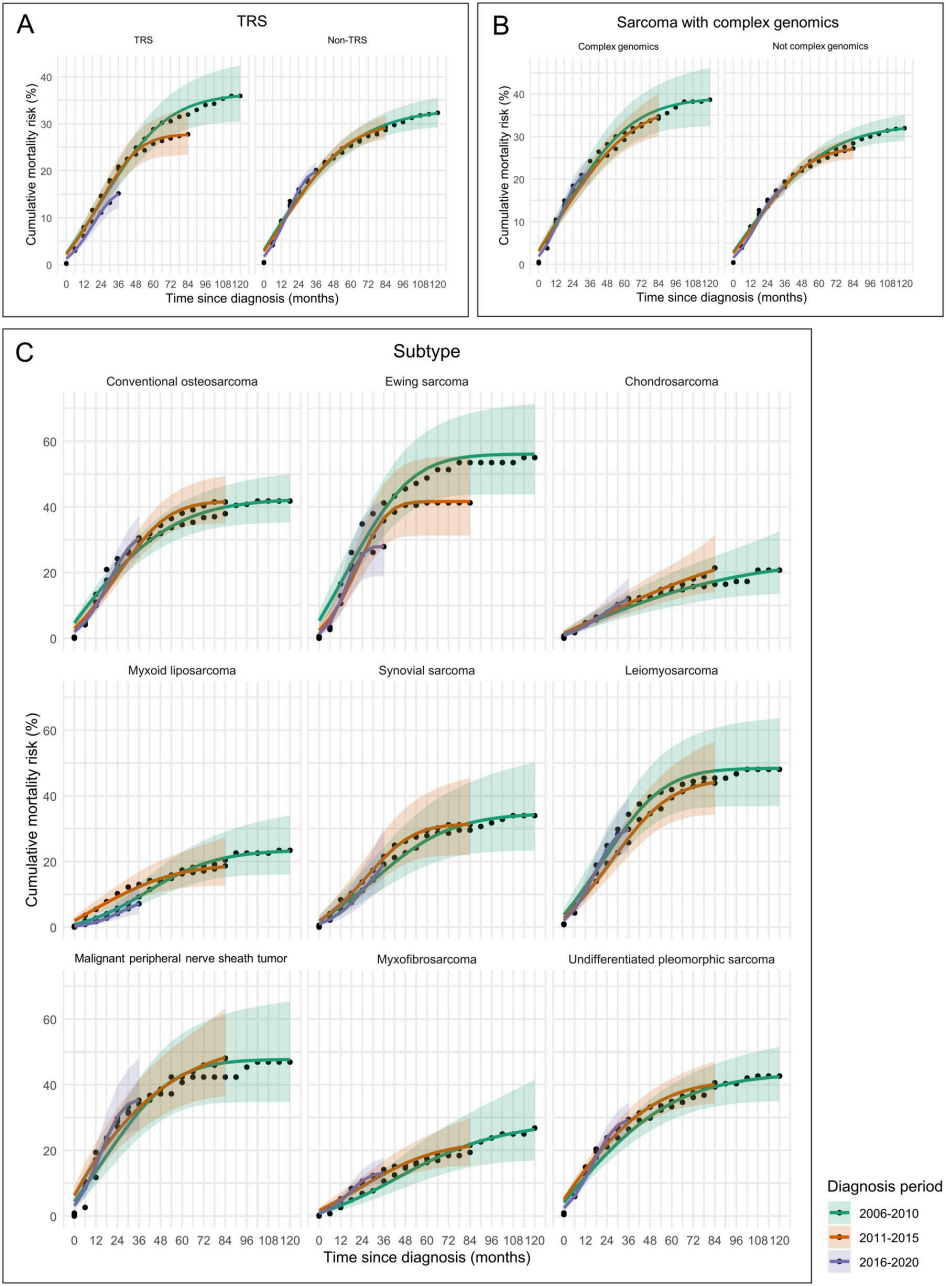
Trends in cumulative mortality risk by histological subtype. Translocation-related sarcomas showed a tendency toward a lower cumulative mortality risk in more recent diagnostic periods **(A)**. No consistent trend was observed when classified according to complex genomic status **(B)**. Among the major histological subtypes, only Ewing sarcoma showed a decreasing trend in cumulative mortality risk **(C)**. Abbreviation: TRS = translocation-related sarcoma.

Given the distinct clinical characteristics of translocation-related sarcomas and nontranslocation-related sarcomas,^2^ we compared their cumulative mortality risks. Translocation-related sarcomas showed a decreasing mortality trend in the more recent diagnostic periods (Figure 4, A). In contrast, nontranslocation-related sarcomas did not exhibit clear changes across the diagnostic periods. Sarcomas with complex genomics, which are generally associated with poor prognosis,^11^ had a higher mortality risk than other sarcomas; however, no consistent trend was observed across the diagnostic periods (Figure 4, B). Among individual subtypes, Ewing sarcoma was the only one that showed a consistent decline in mortality risk over time (Figure 4, C), whereas the other subtypes displayed no clear trends and mortality risks remained relatively stable.

### Sensitivity analysis

Although only 1485 (7.5%) patients had any missing values (Figure S1), we performed multiple imputations, including variables not directly involved in mortality estimation, to assess the robustness of the findings. The results were consistent with the main analysis, and the findings for all patients are presented in Figure S2. Next, a Kaplan–Meier survival analysis was performed. By not setting a follow-up period cutoff, long-term prognosis could be evaluated, particularly for the 2016-2020 group (Figures S3-8). These results are consistent with those of the main analysis across all subgroups. Notably, for Ewing sarcoma, the declining mortality trend observed in the main analysis was more pronounced (Figure S8). Finally, competing risk analysis was conducted using the cumulative incidence function, focusing on tumor-related mortality.^13^ These results are consistent with those of the main analysis and other sensitivity analyses (Figures S9-14).

### Temporal trends in limb amputation

Among patients with limb primaries, the proportion of those who underwent amputation was 8.0% (252 of 3138) in 2006-2010, 7.9% (340 of 4310) in 2011-2015, and 7.1% (351 of 4954) in 2016-2020.

In logistic regression analysis adjusting for observation time, the odds ratios (ORs) of amputation were not statistically significantly different between 2006-2010 and 2011-2015 (OR = 0.94, 95% CI = 0.79 to 1.11) but were significantly lower in 2016-2020 compared with 2006-2010 (OR = 0.77, 95% CI = 0.65 to 0.92). These findings suggest a temporal decrease in the amputation for limb sarcoma in the most recent diagnosis period.

## Discussion

This study examined the changes in the mortality of patients with sarcomas over the past 15 years using one of the largest nationwide bone and soft tissue tumor registries. Overall, mortality showed no major changes; however, a declining trend was observed for Ewing sarcoma. In addition, the proportion of patients with limb primaries who underwent amputation decreased statistically significantly in the most recent diagnosis period, suggesting a shift toward limb-salvage procedures over time.

Some studies have examined temporal trends in cancer prognosis.^3-8^^,^^12^^,^^14-17^ Several studies have focused on osteosarcoma.^3-5^ Compared with decades ago, the prognosis of pediatric osteosarcoma has improved; however, recent trends remain insufficiently explored. Most reports on soft tissue sarcomas are outdated, with the most recent study covering patients diagnosed up to 2014.^6^ A previous study was limited to patients with distant metastases at the initial diagnosis, and its subtype analysis broadly categorized patients into liposarcoma, leiomyosarcoma, and others.^6^ One small-scale study covering cases up to 2010 did not conduct subtype-specific analyses.^8^ The most comprehensive study to date has focused on resected soft tissue sarcomas of the extremities; however, it included only patients diagnosed up to 2001 and was limited to the following subtypes: fibrosarcoma, liposarcoma, leiomyosarcoma, malignant fibrous histiocytoma, and synovial sarcoma.^7^ A small-scale study reported improvements in sarcoma prognosis,^8^ whereas larger studies reported only limited improvement.^6^^,^^7^

In contrast to prior studies that analyzed fewer than 5000 participants,^3-8^ this study incorporated a substantially larger cohort, allowing for a more detailed examination of the prognostic trends in bone and soft tissue sarcomas. In addition, it provides a more comprehensive analysis by evaluating previously unexamined subtypes, treatment modalities, and clinical factors. Furthermore, by including patients diagnosed after 2010, when advancements in chemotherapy accelerated, this study reflects the most recent trends. For soft tissue sarcomas, the prognosis did not improve, which is consistent with previous studies that analyzed cases from the 2010s.^6^ For osteosarcoma, previous reports have suggested improved prognosis in pediatric patients,^3^^,^^5^ but this study did not confirm this trend. However, the magnitude of this improvement has been shrinking over time, with only minor increases observed from 1990 to 2016.^3^ Given that this study evaluated more recent data, its findings do not contradict those of previous studies.

Ewing sarcoma was the only subtype that showed a consistent trend toward improved prognosis. Notably, it is the only sarcoma subtype for which the standard treatment, including first-line therapy, has been modified since 2006. A key advancement has been the introduction of interval-compressed chemotherapy with vincristine, doxorubicin, and cyclophosphamide, alternating with ifosfamide and etoposide.^18^ Additionally, the efficacy of high-dose chemotherapy with autologous stem cell transplantation has been reported in locally advanced, high-risk patients, providing new treatment options for advanced disease.^19^ These therapeutic advancements have likely contributed to the observed improvements in Ewing sarcoma prognosis. A slight improvement was also suggested in pediatric and translocation-related sarcoma patients, which may reflect the influence of Ewing sarcoma outcomes.

In contrast, for osteosarcoma and other bone and soft tissue sarcomas, there have been no major changes in the standard first-line treatment over the past 15 years. Among the drugs introduced in the past decade, pazopanib and trabectedin demonstrated progression-free survival benefits compared with dacarbazine but did not improve overall survival.^20^^,^^21^ Eribulin extended the overall survival, but the difference was limited to 1.3 months.^22^ Given these results, doxorubicin-containing regimens, such as doxorubicin plus ifosfamide (AI therapy), remain the first-line standard chemotherapy for advanced or resectable high-grade nonround cell soft tissue sarcomas, which constitute the majority of patients.^23^ Most bone and soft tissue sarcomas show little change with standard therapies, which may explain the lack of meaningful improvements in prognosis. In other words, new drugs introduced as second-line or later treatments did not have a substantial effect on overall survival trends in sarcomas.

Although the overall prognosis of sarcomas showed no marked improvement, the proportion of patients with limb primaries who underwent amputation decreased after 2016, suggesting a potential improvement in patients’ activities of daily living. However, because detailed information on treatment modalities was not available in this study, it remains unclear whether this reduction in amputation reflects advances in surgical and radiotherapeutic local control techniques or improvements in systemic disease control through chemotherapy.

Most of the clinical trials that were conducted to evaluate new therapies for sarcomas failed to demonstrate enough efficacy.^24-32^ However, several clinical trials are still ongoing,^33-35^ and promising treatment options are emerging for some subtypes.^36^^,^^37^ New treatment strategies that fundamentally alter the current standard of care are required to improve the prognosis of patients with sarcomas. The results of future clinical trials will be crucial in shaping advancements in the treatment of sarcomas.

This study had several limitations. First, the diagnoses relied on data provided by individual institutions, which may have introduced variability in the accuracy. However, to ensure diagnostic reliability, only pathologically confirmed patients were included in the analysis. Nevertheless, sarcoma diagnosis often requires assessment by pathologists with specific expertise in sarcomas and the integration of multiple ancillary techniques, including immunohistochemistry, fluorescence in situ hybridization, and sequencing-based genetic testing. In particular, sarcomas with complex genomics are frequently classified histologically as pleomorphic sarcomas and lack distinctive morphologic features, making accurate diagnosis challenging. Therefore, diagnostic heterogeneity and potential misclassification may have been greater for sarcomas with complex genomics than for sarcomas with more characteristic histologic or molecular profiles. Second, improved medical access and wider availability of diagnostic imaging may have led to earlier detection, potentially causing lead-time bias. However, unlike carcinomas, there are no established screening programs for sarcomas, which suggests that this effect is minimal. Third, this study did not include patients treated at nonregistered institutions, indicating that it may not fully represent all sarcoma patients in Japan. However, sarcoma treatment requires a high level of specialization, and most patients are managed at registry-affiliated institutions, making the number of unregistered patients very small.

This study aimed to update the trends in sarcoma prognosis over time. Despite an increasing number of treatment options for bone and soft tissue sarcomas in recent years, improvements in survival have been limited, except for Ewing sarcoma. The development of breakthrough therapies is essential for achieving meaningful advancements in the treatment of sarcoma.

## Supplementary Material

pkag001_Supplementary_Data

## Data Availability

The dataset used in this study was provided by the Bone and Soft Tissue Tumor Committee of the Japanese Orthopaedic Association and cannot be shared because of institutional regulations. The code used for data analysis is available from the corresponding author upon reasonable request.
