# Supplementary material for: Bone and soft tissue sarcoma mortality in 19 811 patients diagnosed in Japan, 2006-2020
Source: JNCI Cancer Spectr. 2026 Jan 7;10(1):pkag001. doi: 10.1093/jncics/pkag001 (PMC12901746; doi:10.1093/jncics/pkag001)
Supplement: pkag001_Supplementary_Data [file pkag001_supplementary_data.zip › Supplemental_materials.pdf]

Supplementary materials

Bone and soft tissue sarcoma mortality in 19,811 patients diagnosed in Japan, 2006–2020

Contents

**Table S1**	Definition of translocation-related sarcomas
**Table S2**	Definition of sarcoma with complex genomics
**Figure S1**	Visualization of missing data
**Figure S2**	Trends in cumulative mortality risk by overall cohort using multiple imputation
**Figure S3**	Trends in cumulative mortality by overall cohort using Kaplan–Meier method
**Figure S4**	Trends in cumulative mortality by age using Kaplan–Meier method
**Figure S5**	Trends in cumulative mortality by clinical factors using Kaplan–Meier method
**Figure S6**	Trends in cumulative mortality by treatment modality using Kaplan–Meier method
**Figure S7**	Trends in cumulative mortality by subtype categories using Kaplan–Meier method
**Figure S8**	Trends in cumulative mortality by histological subtype using Kaplan–Meier method
**Figure S9**	Trends in cumulative mortality by overall cohort using cumulative incidence function
**Figure S10**	Trends in cumulative mortality by age using cumulative incidence function
**Figure S11**	Trends in cumulative mortality by clinical factors using cumulative incidence function
**Figure S12**	Trends in cumulative mortality by treatment modality using cumulative incidence function
**Figure S13**	Trends in cumulative mortality by subtype categories using cumulative incidence function
**Figure S14**	Trends in cumulative mortality by histological subtype using cumulative incidence function

Table S1. Definition of translocation-related sarcomas

TRS
Alveolar rhabdomyosarcoma
Alveolar soft part sarcoma
CIC rearranged sarcoma
Clear cell sarcoma
Dermatofibrosarcoma protuberans
Desmoplastic small round cell tumor
Epithelioid hemangioendothelioma
Ewing sarcoma
Extraskeletal myxoid chondrosarcoma
Inflammatory myofibroblastic tumor
Infantile fibrosarcoma
Low-grade fibromyxoid sarcoma
Myxoid liposarcoma
Mesenchymal chondrosarcoma
NTRK rearranged spindle cell neoplasm
Sarcoma with *BCOR* genetic alterations
Sclerosing epithelioid fibrosarcoma
Solitary fibrous tumor
Synovial sarcoma

Abbreviation: TRS, translocation-related sarcomas

Table S2. Definition of sarcomas with complex genomics

Sarcomas with complex genomics
Dedifferentiated liposarcoma
Malignant peripheral nerve sheath tumor
Leiomyosarcoma
Pleomorphic rhabdomyosarcoma
Myxofibrosarcoma
Pleomorphic liposarcoma

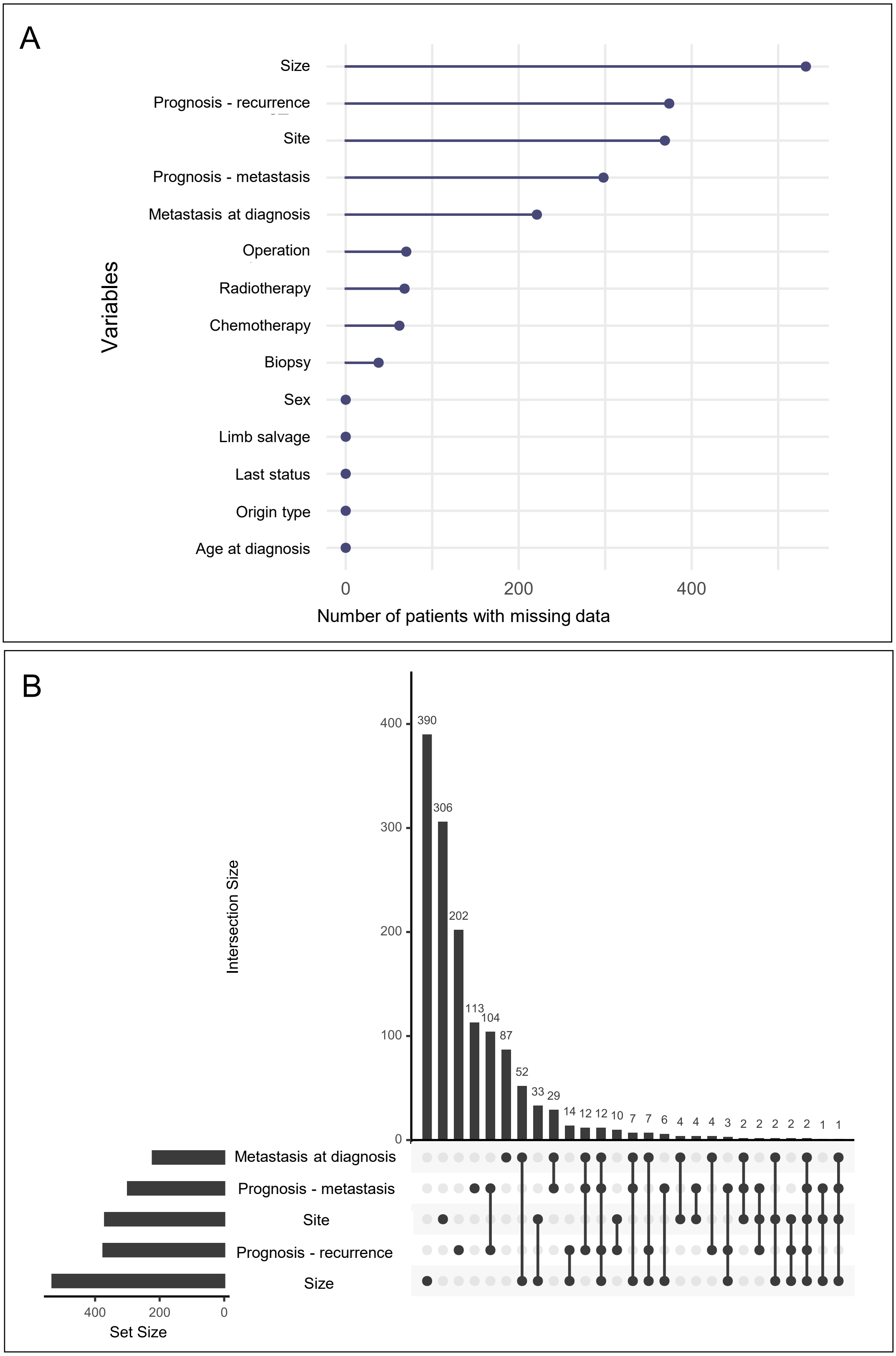

Figure S1. Visualization of missing data

The distribution of missing values across variables (**A**) and visualization of the association between missing data and the main factors (**B**).

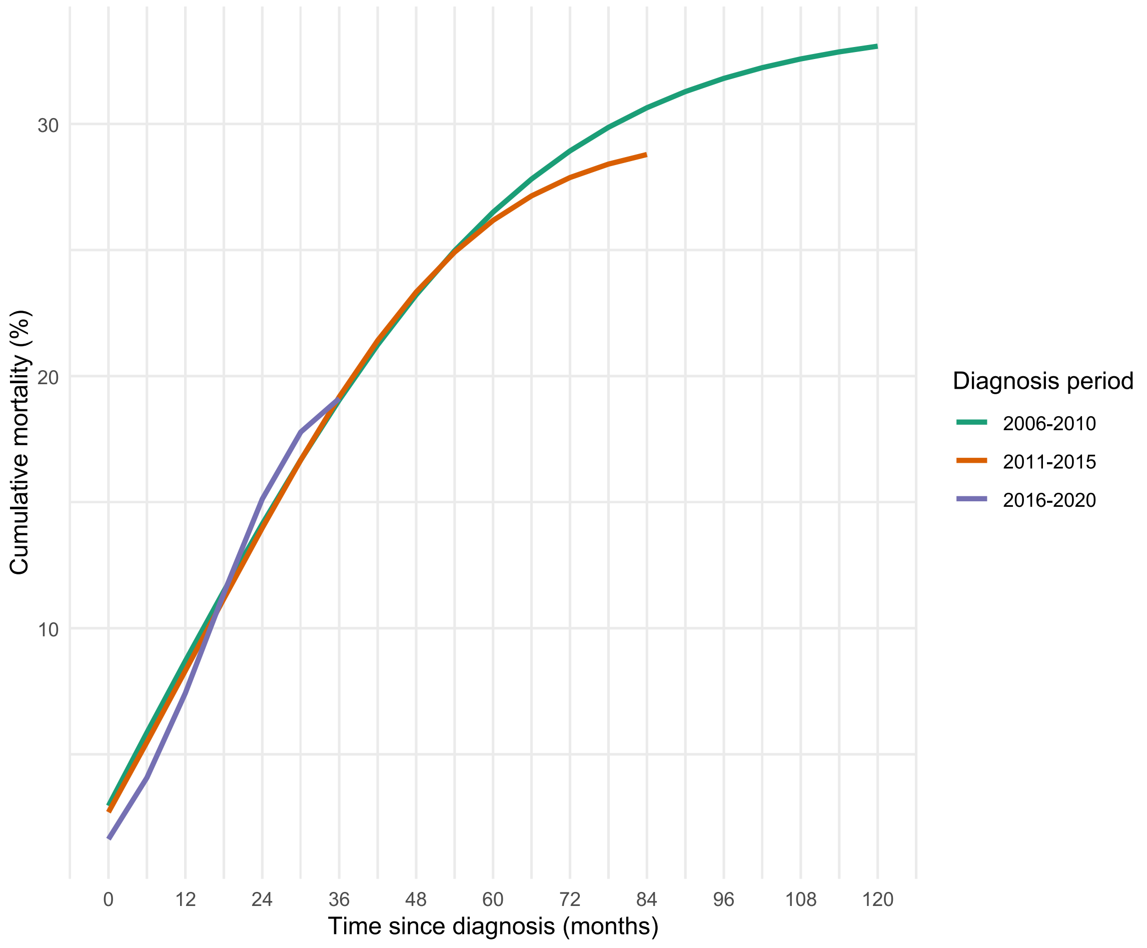

Figure S2. Trends in cumulative mortality risk by overall cohort using multiple imputation

Multiple imputations were performed using the *mice* package in R, including variables not directly involved in mortality risk estimation. Poisson regression was applied to the imputed dataset in the same manner as in the main analysis, and the results were combined using Rubin's rules for visualization. These results are consistent with those of the main analysis, showing no clear differences across the diagnostic periods.

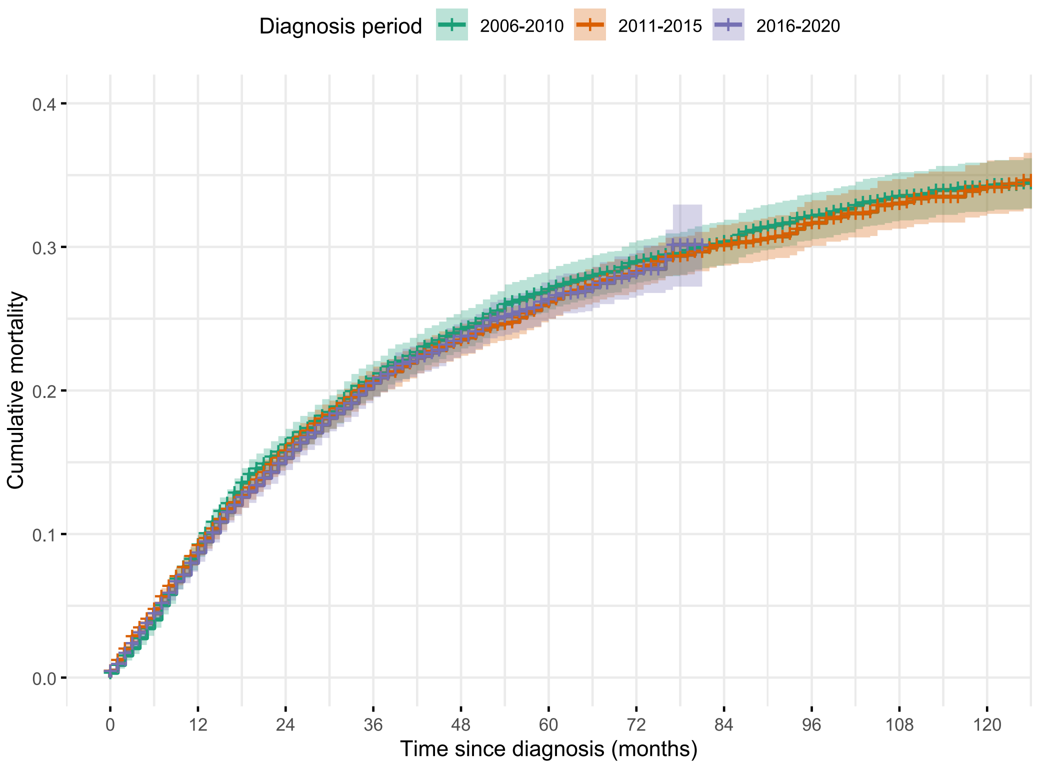

Number at risk

Diagnosis period	0	12	24	36	48	60	72	84	96	108	120
2006-2010	4687	3956	3412	2921	2381	2133	1654	1249	1137	1025	806
2011-2015	6708	5564	4722	3876	3213	2655	1545	733	641	579	436
2016-2020	8091	6554	5269	3042	1514	1199	365	0	0	0	0

Time since diagnosis (months)

Figure S3. Trends in cumulative mortality by overall cohort using Kaplan–Meier method

The Kaplan–Meier method was used to visualize cumulative mortality as 1 - survival probability. A complete case analysis was performed using the entire follow-up period. This approach allowed for a long-term estimation, particularly in the 2016–2020 group. The results were consistent with those of the main analysis, showing no clear differences in cumulative mortality across the diagnostic periods.

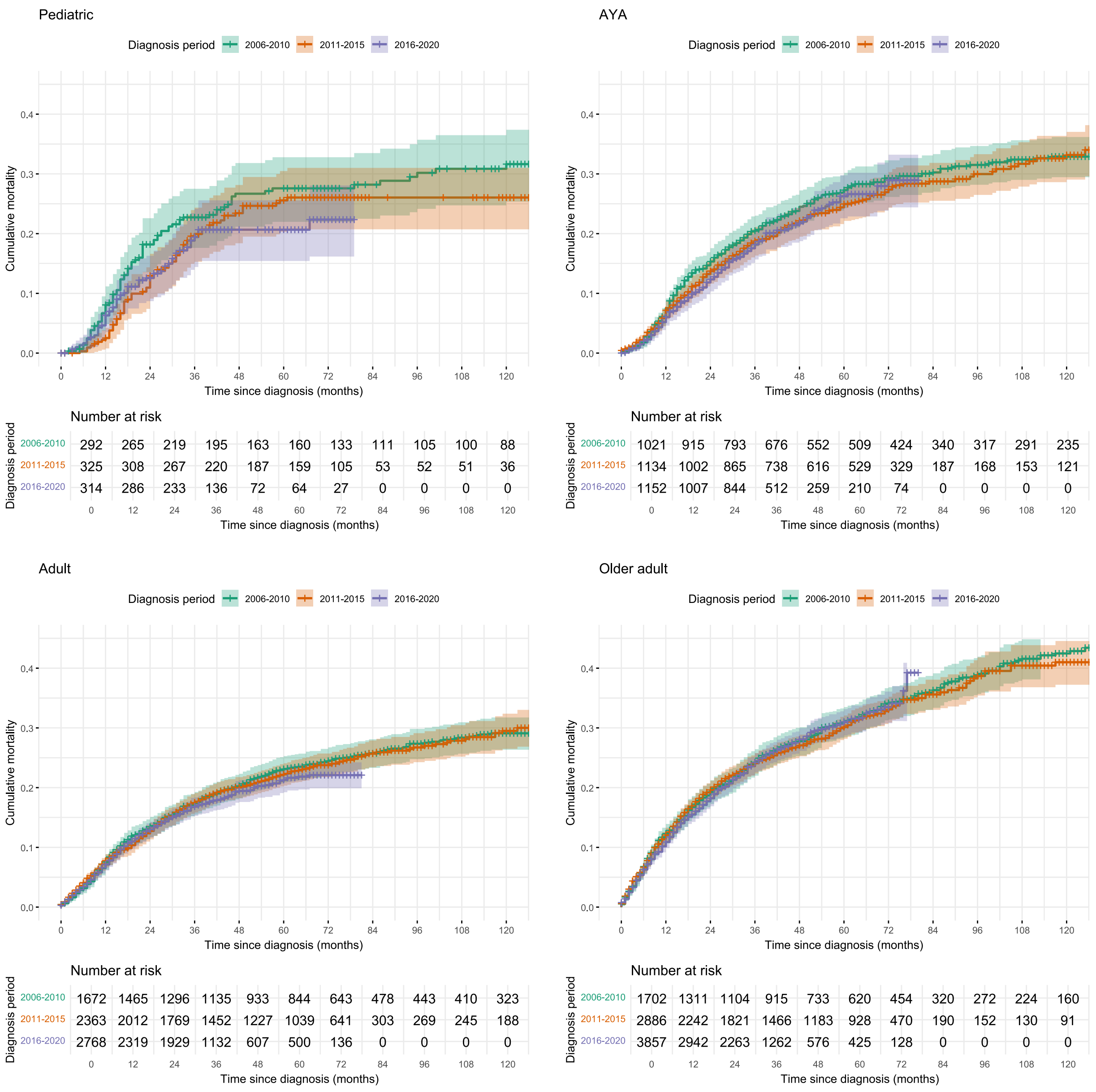

Figure S4. Trends in cumulative mortality by age using Kaplan–Meier method

Separate models were created for each age category. In the pediatric group, cumulative mortality tended to be lower in patients diagnosed in 2011 and later than in those diagnosed before 2011.

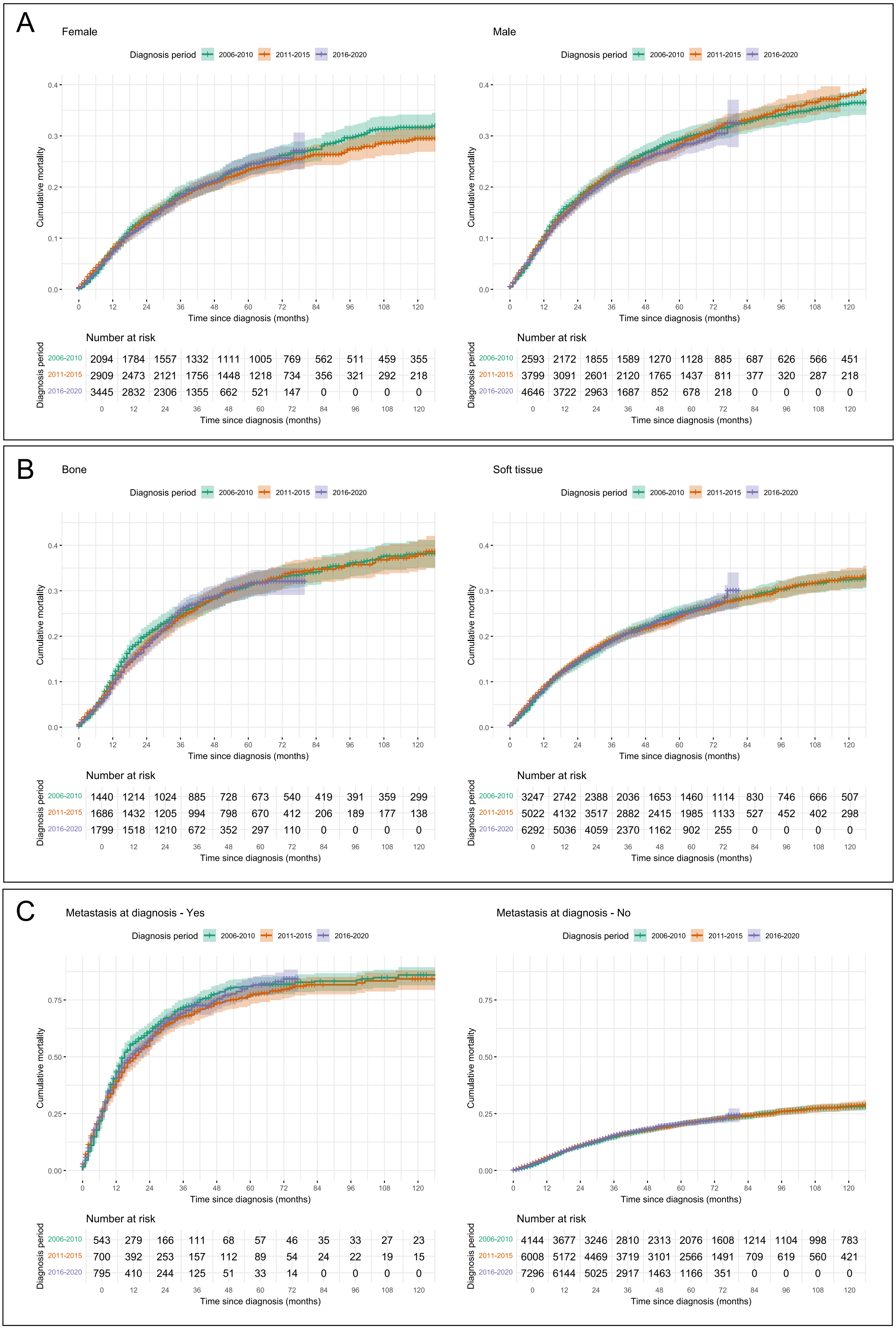

Figure S5. Trends in cumulative mortality by clinical factors using Kaplan–Meier method

Separate models were created and visualized for sex (**A**), origin type (bone or soft tissue) (**B**), and the presence of metastasis at diagnosis (**C**). Consistent with the main analysis, no clear trends in cumulative mortality were observed across diagnostic periods.

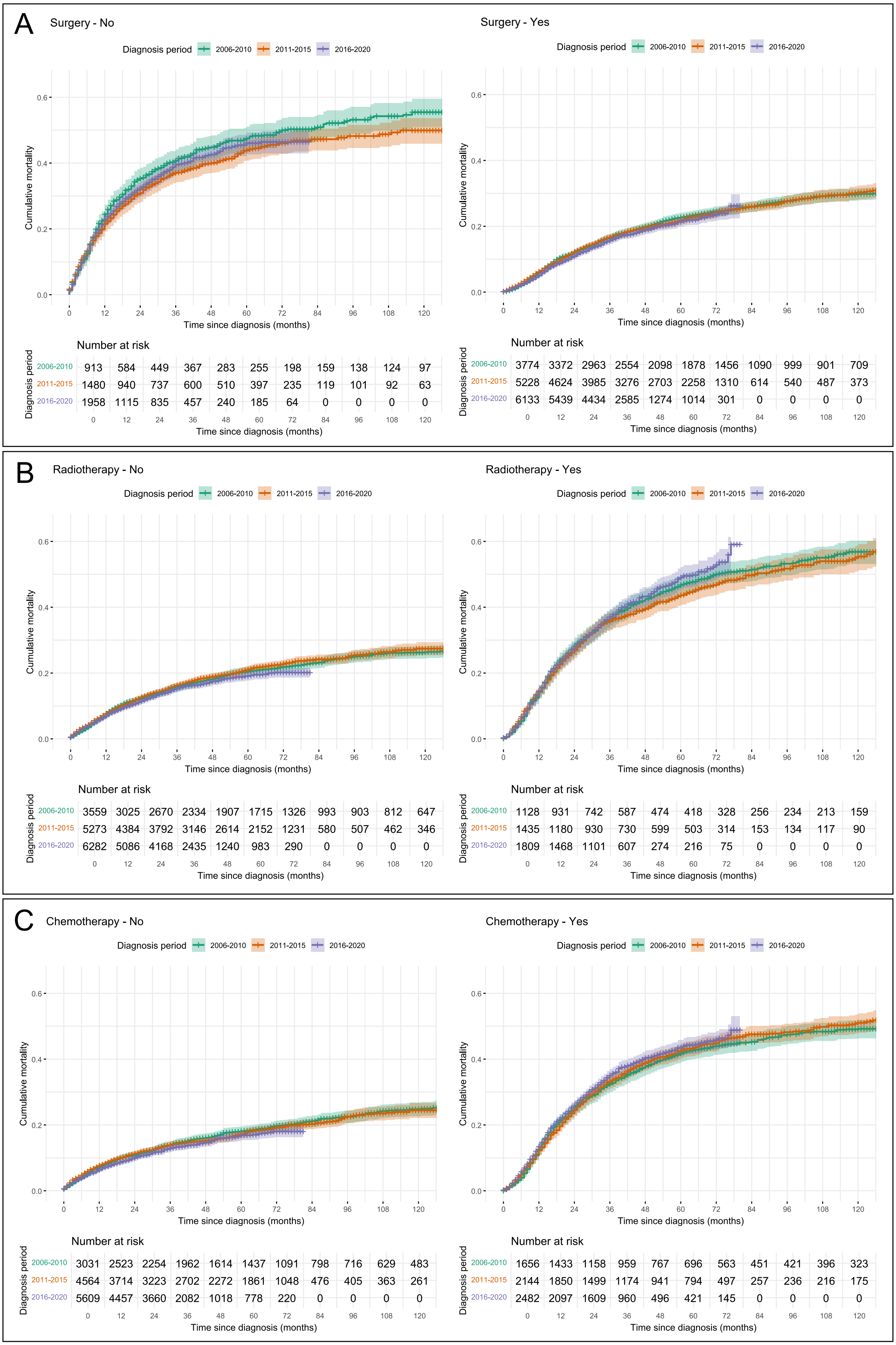

Figure S6. Trends in cumulative mortality by treatment modality using Kaplan–Meier method

Separate models were created and visualized for surgery (**A**), radiotherapy (**B**), and chemotherapy (**C**). Consistent with the main analysis, no clear trends in cumulative mortality were observed across diagnostic periods.

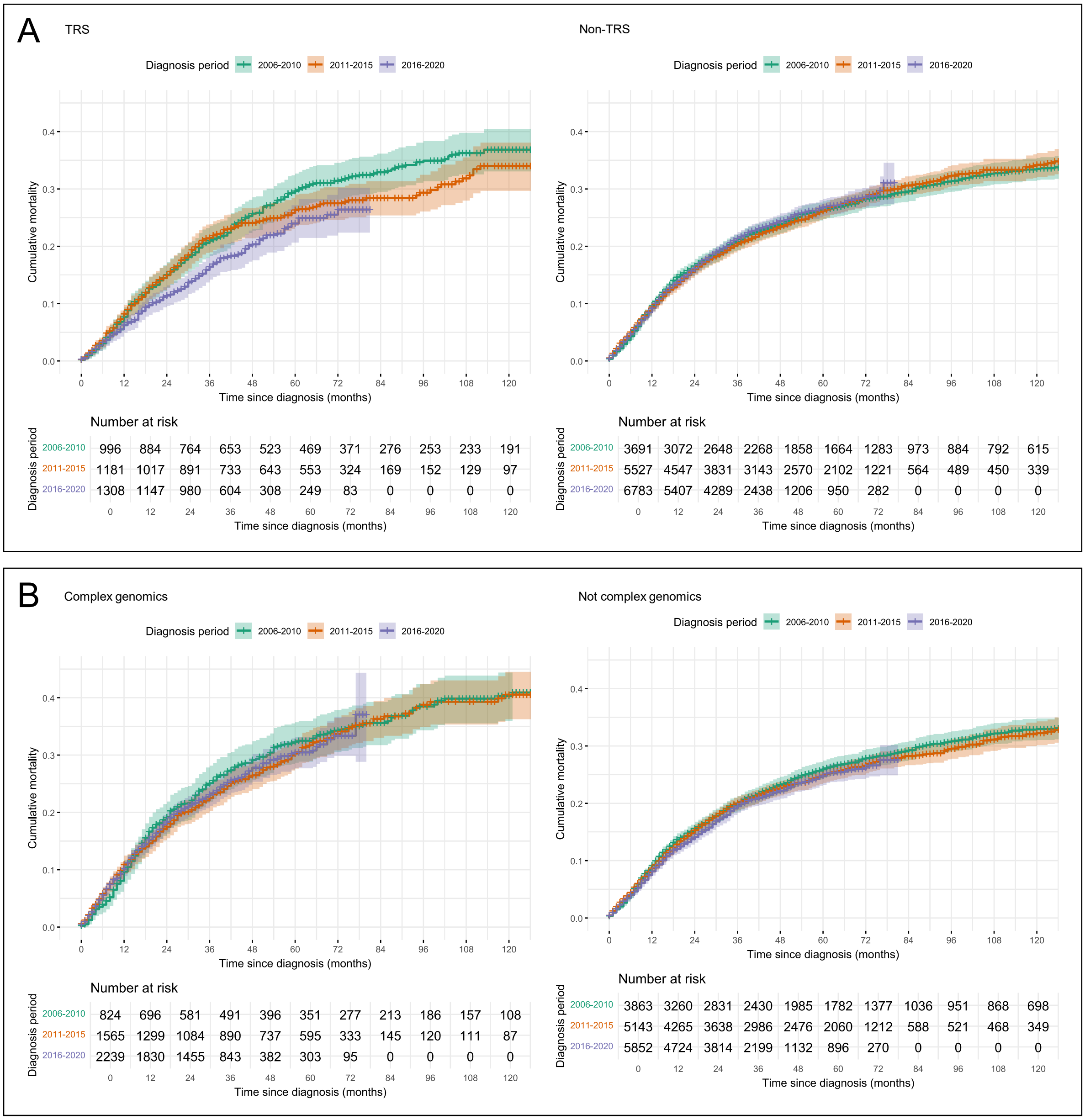

Figure S7. Trends in cumulative mortality by TRS and complex genomics using Kaplan–Meier method

Cumulative mortality was visualized for translocation-related sarcomas (TRS) versus non-TRS (**A**) and sarcomas with complex genomics versus other sarcomas (**B**). A decreasing trend in the cumulative mortality was observed only in the TRS group.

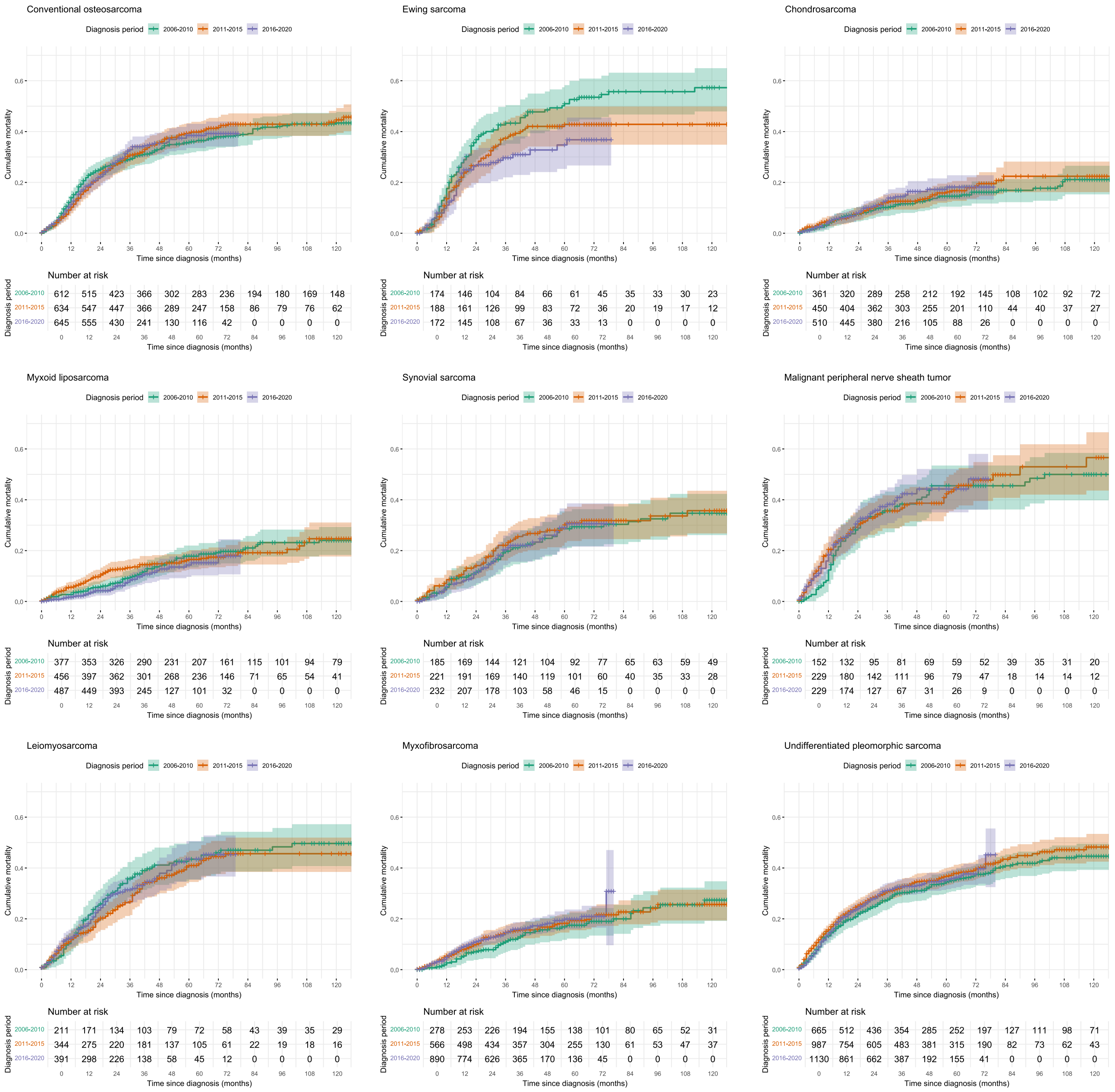

Figure S8. Trends in cumulative mortality by histological subtype using Kaplan–Meier method

Separate models were created for the major histological subtypes, and cumulative mortality was visualized. Consistent with the main analysis, only Ewing sarcoma showed a decreasing trend in cumulative mortality over time. Using the entire follow-up period allowed for a long-term prognosis estimation, making this trend more evident.

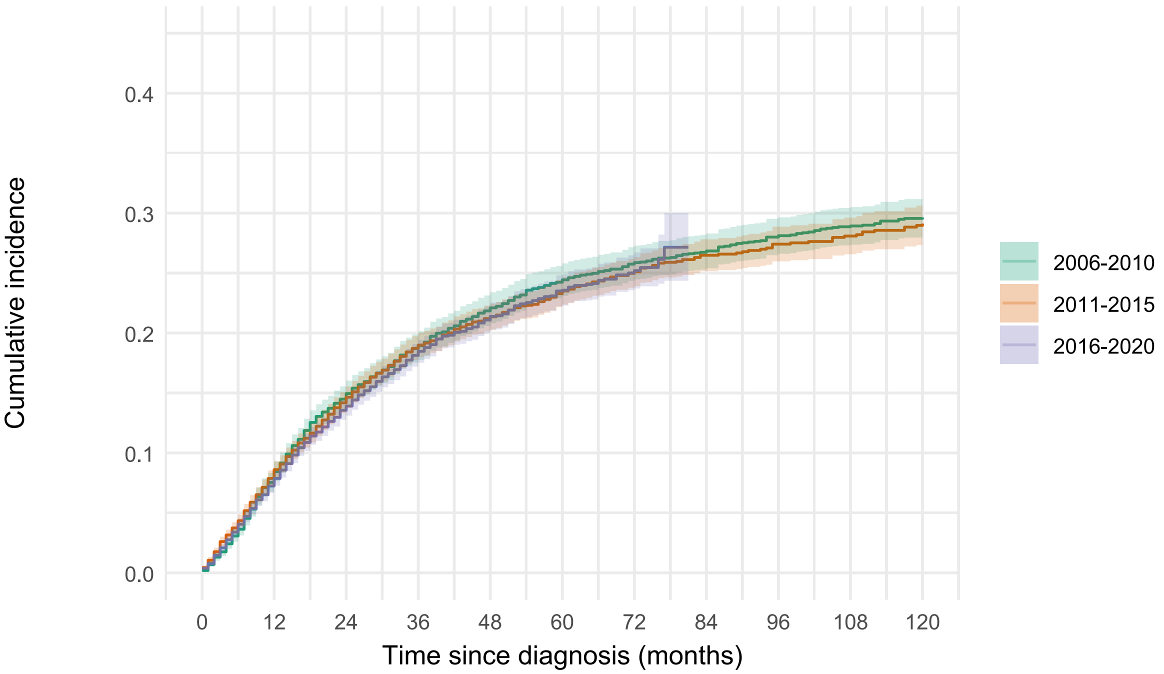

At Risk

2006-2010	4687	3956	3412	2921	2381	2133	1654	1249	1137	1025	806
2011-2015	6708	5564	4722	3876	3213	2655	1545	733	641	579	436
2016-2020	8091	6554	5269	3042	1514	1199	365	0	0	0	0

Events

2006-2010	9	374	647	802	908	979	1016	1035	1057	1070	1080
2011-2015	29	542	892	1120	1225	1309	1354	1375	1384	1390	1398
2016-2020	30	583	988	1220	1296	1336	1353	1356	1356	1356	1356

Figure S9. Trends in cumulative mortality by overall cohort using cumulative incidence function

A competing risk analysis was performed using the cumulative incidence function, with death from other causes considered a competing risk for death from the disease. A complete case analysis was conducted using the entire follow-up period. These results are consistent with those of the main analysis, showing no clear trends in disease-specific mortality across the diagnostic periods.

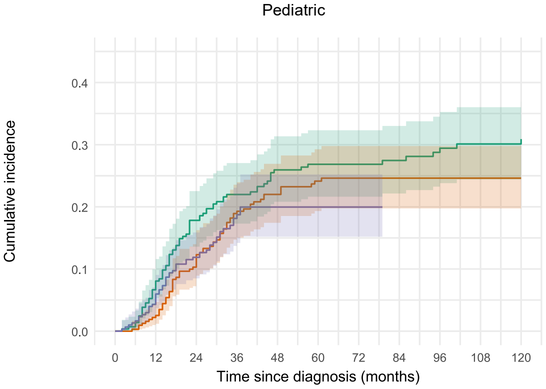

At Risk											
2006-2010	292	265	219	195	163	160	133	111	105	100	88
2011-2015	325	308	267	220	187	159	105	53	52	51	36
2016-2020	314	286	233	136	72	64	27	0	0	0	0

Events											
2006-2010	0	23	50	61	70	72	72	73	76	77	78
2011-2015	0	8	38	58	65	70	71	71	71	71	71
2016-2020	0	18	35	50	52	52	52	52	52	52	52

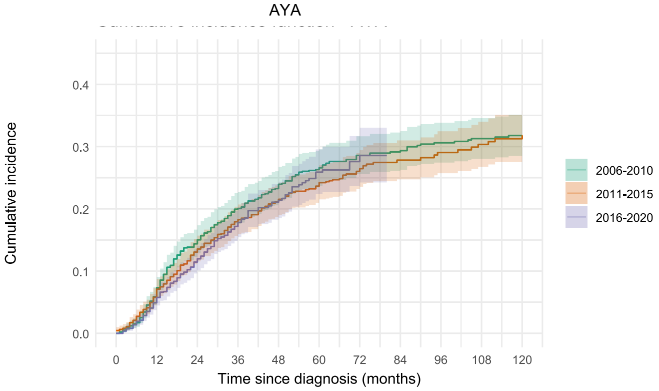

At Risk											
2006-2010	1021	915	793	676	552	509	424	340	317	291	235
2011-2015	1134	1002	865	738	616	529	329	187	168	153	121
2016-2020	1152	1007	844	512	259	210	74	0	0	0	0

Events											
2006-2010	0	72	146	191	221	240	253	257	263	266	268
2011-2015	5	77	143	188	215	235	247	252	255	258	261
2016-2020	0	62	125	172	188	202	206	206	206	206	206

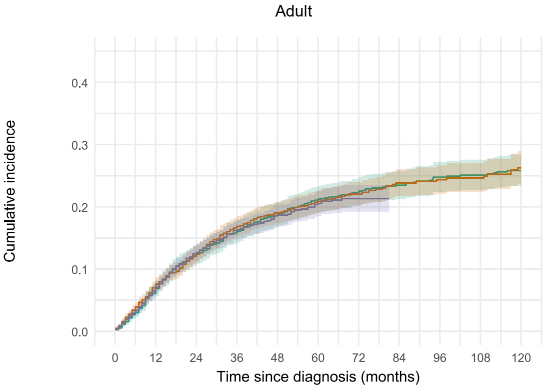

At Risk											
2006-2010	1672	1465	1296	1135	933	844	643	478	443	410	323
2011-2015	2363	2012	1769	1452	1227	1039	641	303	269	245	188
2016-2020	2768	2319	1929	1132	607	500	136	0	0	0	0

Events											
2006-2010	5	110	195	247	281	309	321	328	336	338	342
2011-2015	10	169	270	354	389	416	429	439	441	442	447
2016-2020	7	180	313	379	401	414	418	418	418	418	418

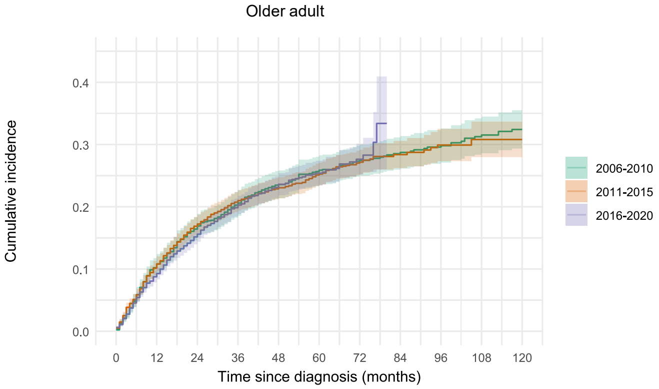

At Risk											
2006-2010	1702	1311	1104	915	733	620	454	320	272	224	160
2011-2015	2886	2242	1821	1466	1183	928	470	190	152	130	91
2016-2020	3857	2942	2263	1262	576	425	128	0	0	0	0

Events											
2006-2010	4	169	256	303	336	358	370	377	382	389	392
2011-2015	14	288	441	520	556	588	607	613	617	619	619
2016-2020	23	323	515	619	655	668	677	680	680	680	680

Figure S10. Trends in cumulative mortality by age using cumulative incidence function

Separate models were created for each age category. In the pediatric group, the cumulative incidence tended to be lower in patients diagnosed in 2011 and later than in those diagnosed before 2011.

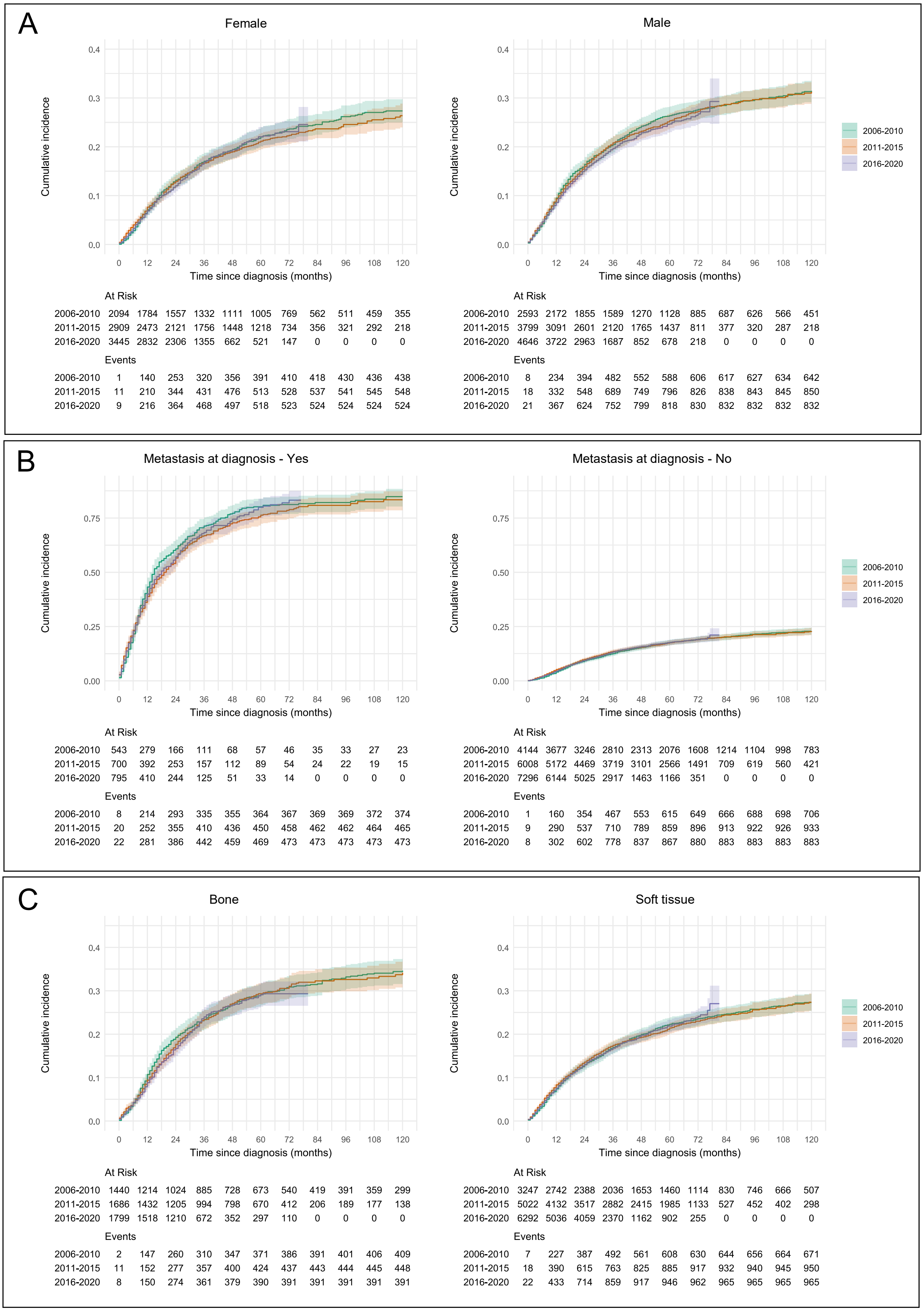

A — Female

At Risk	0	12	24	36	48	60	72	84	96	108	120
2006-2010	2094	1784	1557	1332	1111	1005	769	562	511	459	355
2011-2015	2909	2473	2121	1756	1448	1218	734	356	321	292	218
2016-2020	3445	2832	2306	1355	662	521	147	0	0	0	0

Events	0	12	24	36	48	60	72	84	96	108	120
2006-2010	1	140	253	320	356	391	410	418	430	436	438
2011-2015	11	210	344	431	476	513	528	537	541	545	548
2016-2020	9	216	364	468	497	518	523	524	524	524	524

A — Male

At Risk	0	12	24	36	48	60	72	84	96	108	120
2006-2010	2593	2172	1855	1589	1270	1128	885	687	626	566	451
2011-2015	3799	3091	2601	2120	1765	1437	811	377	320	287	218
2016-2020	4646	3722	2963	1687	852	678	218	0	0	0	0

Events	0	12	24	36	48	60	72	84	96	108	120
2006-2010	8	234	394	482	552	588	606	617	627	634	642
2011-2015	18	332	548	689	749	796	826	838	843	845	850
2016-2020	21	367	624	752	799	818	830	832	832	832	832

B — Metastasis at diagnosis - Yes

At Risk	0	12	24	36	48	60	72	84	96	108	120
2006-2010	543	279	166	111	68	57	46	35	33	27	23
2011-2015	700	392	253	157	112	89	54	24	22	19	15
2016-2020	795	410	244	125	51	33	14	0	0	0	0

Events	0	12	24	36	48	60	72	84	96	108	120
2006-2010	8	214	293	335	355	364	367	369	369	372	374
2011-2015	20	252	355	410	436	450	458	462	462	464	465
2016-2020	22	281	386	442	459	469	473	473	473	473	473

B — Metastasis at diagnosis - No

At Risk	0	12	24	36	48	60	72	84	96	108	120
2006-2010	4144	3677	3246	2810	2313	2076	1608	1214	1104	998	783
2011-2015	6008	5172	4469	3719	3101	2566	1491	709	619	560	421
2016-2020	7296	6144	5025	2917	1463	1166	351	0	0	0	0

Events	0	12	24	36	48	60	72	84	96	108	120
2006-2010	1	160	354	467	553	615	649	666	688	698	706
2011-2015	9	290	537	710	789	859	896	913	922	926	933
2016-2020	8	302	602	778	837	867	880	883	883	883	883

C — Bone

At Risk	0	12	24	36	48	60	72	84	96	108	120
2006-2010	1440	1214	1024	885	728	673	540	419	391	359	299
2011-2015	1686	1432	1205	994	798	670	412	206	189	177	138
2016-2020	1799	1518	1210	672	352	297	110	0	0	0	0

Events	0	12	24	36	48	60	72	84	96	108	120
2006-2010	2	147	260	310	347	371	386	391	401	406	409
2011-2015	11	152	277	357	400	424	437	443	444	445	448
2016-2020	8	150	274	361	379	390	391	391	391	391	391

C — Soft tissue

At Risk	0	12	24	36	48	60	72	84	96	108	120
2006-2010	3247	2742	2388	2036	1653	1460	1114	830	746	666	507
2011-2015	5022	4132	3517	2882	2415	1985	1133	527	452	402	298
2016-2020	6292	5036	4059	2370	1162	902	255	0	0	0	0

Events	0	12	24	36	48	60	72	84	96	108	120
2006-2010	7	227	387	492	561	608	630	644	656	664	671
2011-2015	18	390	615	763	825	885	917	932	940	945	950
2016-2020	22	433	714	859	917	946	962	965	965	965	965

Figure S11. Trends in cumulative mortality by clinical factors using cumulative incidence function

Separate models were created and visualized for sex (**A**), origin type (bone or soft tissue) (**B**), and the presence of metastasis at diagnosis (**C**). Consistent with the main analysis, no clear trends in the cumulative incidence were observed across the diagnostic periods.

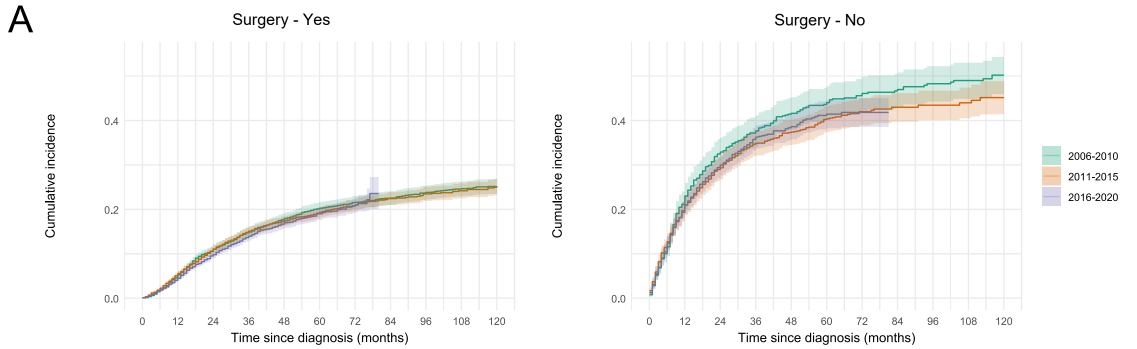

Surgery - Yes

At Risk

2006-2010	3774	3372	2963	2554	2098	1878	1456	1090	999	901	709
2011-2015	5228	4624	3985	3276	2703	2258	1310	614	540	487	373
2016-2020	6133	5439	4434	2585	1274	1014	301	0	0	0	0

Events

2006-2010	2	190	391	515	599	658	686	702	720	731	738
2011-2015	4	278	535	707	790	851	887	905	913	918	924
2016-2020	6	262	547	716	780	809	825	828	828	828	828

Surgery - No

At Risk

2006-2010	913	584	449	367	283	255	198	159	138	124	97
2011-2015	1480	940	737	600	510	397	235	119	101	92	63
2016-2020	1958	1115	835	457	240	185	64	0	0	0	0

Events

2006-2010	7	184	256	287	309	321	330	333	337	339	342
2011-2015	25	264	357	413	435	458	467	470	471	472	474
2016-2020	24	321	441	504	516	527	528	528	528	528	528

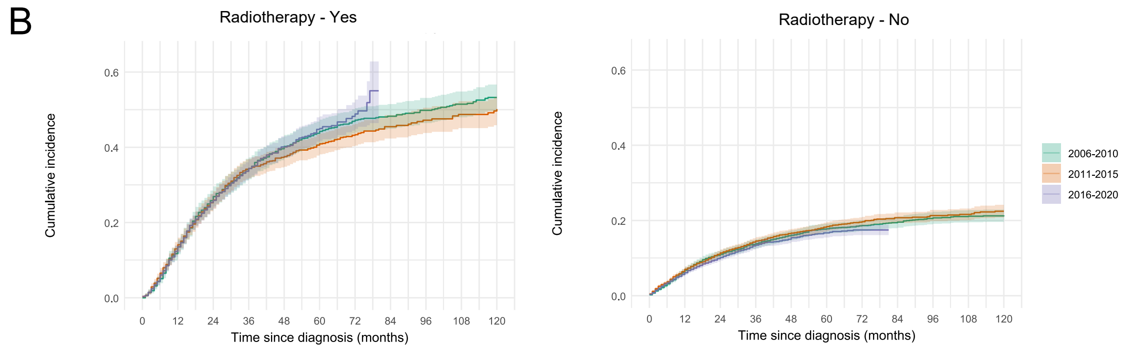

Radiotherapy - Yes

At Risk

2006-2010	1128	931	742	587	474	418	328	256	234	213	159
2011-2015	1435	1180	930	730	599	503	314	153	134	117	90
2016-2020	1809	1468	1101	607	274	216	75	0	0	0	0

Events

2006-2010	0	147	286	359	409	441	462	468	476	484	492
2011-2015	4	196	351	452	485	517	535	544	549	553	556
2016-2020	6	235	434	540	578	600	610	613	613	613	613

Radiotherapy - No

At Risk

2006-2010	3559	3025	2670	2334	1907	1715	1326	993	903	812	647
2011-2015	5273	4384	3792	3146	2614	2152	1231	580	507	462	346
2016-2020	6282	5086	4168	2435	1240	983	290	0	0	0	0

Events

2006-2010	9	227	361	443	499	538	554	567	581	586	588
2011-2015	25	346	541	668	740	792	819	831	835	837	842
2016-2020	24	348	554	680	718	736	743	743	743	743	743

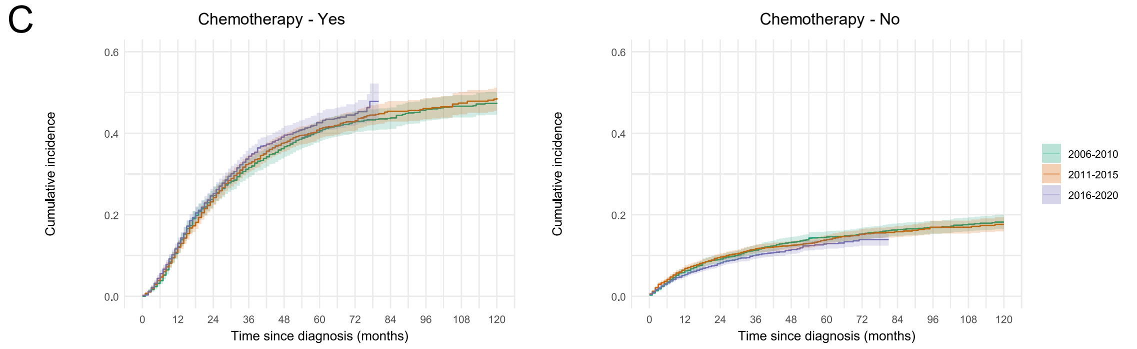

Chemotherapy - Yes

At Risk

2006-2010	1656	1433	1158	959	767	696	563	451	421	396	323
2011-2015	2144	1850	1499	1174	941	794	497	257	236	216	175
2016-2020	2482	2097	1609	960	496	421	145	0	0	0	0

Events

2006-2010	0	198	395	497	563	610	635	643	660	666	672
2011-2015	4	254	498	656	738	788	810	826	829	835	840
2016-2020	4	315	593	762	817	841	853	856	856	856	856

Chemotherapy - No

At Risk

2006-2010	3031	2523	2254	1962	1614	1437	1091	798	716	629	483
2011-2015	4564	3714	3223	2702	2272	1861	1048	476	405	363	261
2016-2020	5609	4457	3660	2082	1018	778	220	0	0	0	0

Events

2006-2010	9	176	252	305	345	369	381	392	397	404	408
2011-2015	25	288	394	464	487	521	544	549	555	555	558
2016-2020	26	268	395	458	479	495	500	500	500	500	500

Figure S12. Trends in cumulative mortality by treatment modality using cumulative incidence function

Separate models were created and visualized for surgery (**A**), radiotherapy (**B**), and chemotherapy (**C**). Consistent with the main analysis, no clear trends in the cumulative incidence were observed across the diagnostic periods.

A

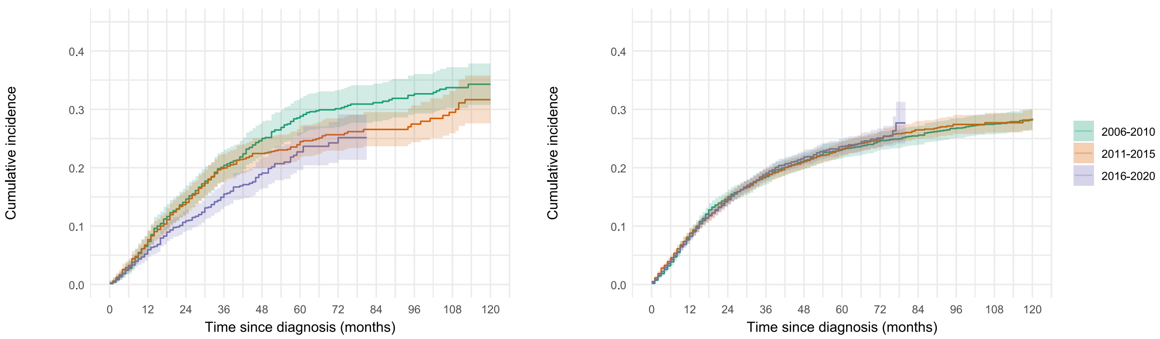

TRS

At Risk

	0	12	24	36	48	60	72	84	96	108	120
2006-2010	996	884	764	653	523	469	371	276	253	233	191
2011-2015	1181	1017	891	733	643	553	324	169	152	129	97
2016-2020	1308	1147	980	604	308	249	83	0	0	0	0

Events

	0	12	24	36	48	60	72	84	96	108	120
2006-2010	2	71	138	188	222	248	256	261	267	271	273
2011-2015	2	86	154	213	235	251	258	261	263	267	271
2016-2020	2	74	132	173	190	203	208	208	208	208	208

Non-TRS

At Risk

	0	12	24	36	48	60	72	84	96	108	120
2006-2010	3691	3072	2648	2268	1858	1664	1283	973	884	792	615
2011-2015	5527	4547	3831	3143	2570	2102	1221	564	489	450	339
2016-2020	6783	5407	4289	2438	1206	950	282	0	0	0	0

Events

	0	12	24	36	48	60	72	84	96	108	120
2006-2010	7	303	509	614	686	731	760	774	790	799	807
2011-2015	27	456	738	907	990	1058	1096	1114	1121	1123	1127
2016-2020	28	509	856	1047	1106	1133	1145	1148	1148	1148	1148

B

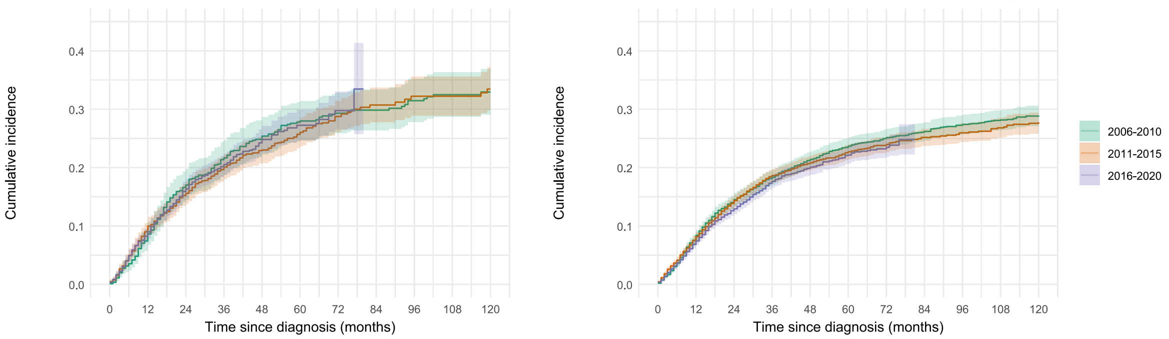

Complex genomics

At Risk

	0	12	24	36	48	60	72	84	96	108	120
2006-2010	824	696	581	491	396	351	277	213	186	157	108
2011-2015	1565	1299	1084	890	737	595	333	145	120	111	87
2016-2020	2239	1830	1455	843	382	303	95	0	0	0	0

Events

	0	12	24	36	48	60	72	84	96	108	120
2006-2010	1	67	129	161	183	197	203	205	210	213	214
2011-2015	7	148	225	280	311	339	357	364	367	367	369
2016-2020	8	188	328	390	420	432	438	439	439	439	439

Not complex genomics

At Risk

	0	12	24	36	48	60	72	84	96	108	120
2006-2010	3863	3260	2831	2430	1985	1782	1377	1036	951	868	698
2011-2015	5143	4265	3638	2986	2476	2060	1212	588	521	468	349
2016-2020	5852	4724	3814	2199	1132	896	270	0	0	0	0

Events

	0	12	24	36	48	60	72	84	96	108	120
2006-2010	8	307	518	641	725	782	813	830	847	857	866
2011-2015	22	394	667	840	914	970	997	1011	1017	1023	1029
2016-2020	22	395	660	830	876	904	915	917	917	917	917

Figure S13. Trends in cumulative mortality by treatment modality using cumulative incidence function

Cumulative incidence was visualized for translocation-related sarcomas (TRS) versus non-TRS (**A**) and sarcomas with complex genomics versus other sarcomas (**B**). A decreasing trend in the cumulative incidence was observed only in the TRS group.

Conventional osteosarcoma

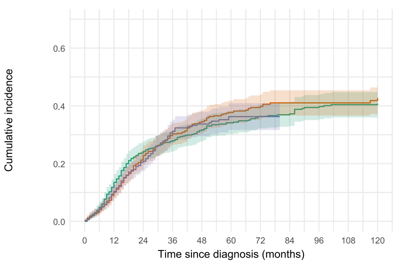

At Risk

	0	12	24	36	48	60	72	84	96	108	120
2006-2010	612	515	423	366	302	283	236	194	180	169	148
2011-2015	634	547	447	366	289	247	158	86	79	76	62
2016-2020	645	555	430	241	130	116	42	0	0	0	0

Events

	0	12	24	36	48	60	72	84	96	108	120
2006-2010	0	80	142	162	180	191	200	203	211	213	214
2011-2015	1	62	136	177	197	211	218	221	221	221	223
2016-2020	2	65	126	174	181	186	186	186	186	186	186

Ewing sarcoma

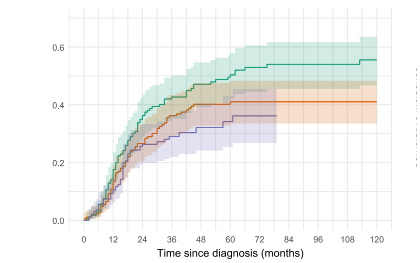

At Risk

	0	12	24	36	48	60	72	84	96	108	120
2006-2010	174	146	104	84	66	61	45	35	33	30	23
2011-2015	188	161	126	99	83	72	36	20	19	17	12
2016-2020	172	145	108	67	36	33	13	0	0	0	0

Events

	0	12	24	36	48	60	72	84	96	108	120
2006-2010	0	30	61	71	77	81	84	85	85	85	86
2011-2015	1	25	48	64	70	71	71	71	71	71	71
2016-2020	0	17	42	45	47	48	49	49	49	49	49

Chondrosarcoma

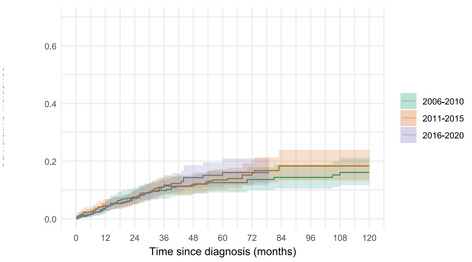

At Risk

	0	12	24	36	48	60	72	84	96	108	120
2006-2010	361	320	289	258	212	192	145	108	102	92	72
2011-2015	450	404	362	303	255	201	110	44	40	37	27
2016-2020	510	445	380	216	105	88	26	0	0	0	0

Events

	0	12	24	36	48	60	72	84	96	108	120
2006-2010	0	14	25	32	38	39	41	42	42	44	44
2011-2015	3	20	30	46	47	52	56	58	58	58	58
2016-2020	2	19	34	48	52	54	54	54	54	54	54

Myxofibrosarcoma

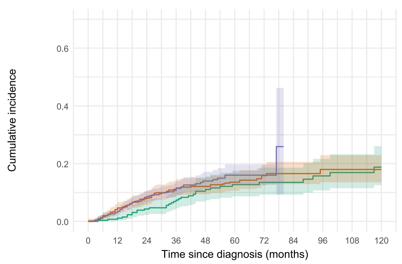

At Risk

	0	12	24	36	48	60	72	84	96	108	120
2006-2010	278	253	226	194	155	138	101	80	65	52	31
2011-2015	566	498	434	357	304	255	130	61	53	47	37
2016-2020	890	774	626	365	170	136	45	0	0	0	0

Events

	0	12	24	36	48	60	72	84	96	108	120
2006-2010	0	3	11	18	25	28	29	29	31	32	33
2011-2015	1	25	42	58	60	65	70	71	72	72	72
2016-2020	0	29	68	83	90	94	94	95	95	95	95

Synovial sarcoma

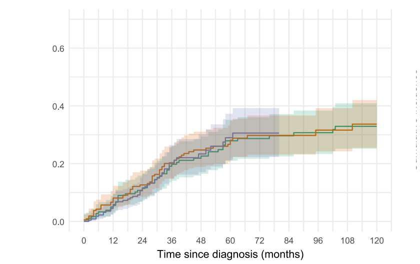

At Risk

	0	12	24	36	48	60	72	84	96	108	120
2006-2010	185	169	144	121	104	92	77	65	63	59	49
2011-2015	221	191	169	140	119	101	60	40	35	33	28
2016-2020	232	207	178	103	58	46	15	0	0	0	0

Events

	0	12	24	36	48	60	72	84	96	108	120
2006-2010	1	10	20	33	37	44	45	46	47	49	49
2011-2015	1	17	26	40	48	53	55	55	56	56	57
2016-2020	0	13	25	39	42	46	47	47	47	47	47

Malignant peripheral nerve sheath tumor

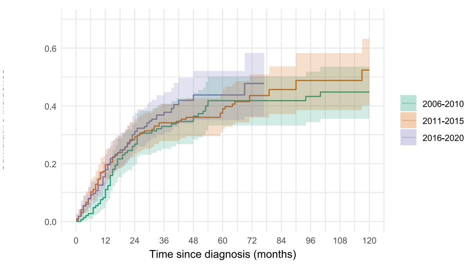

At Risk

	0	12	24	36	48	60	72	84	96	108	120
2006-2010	152	132	95	81	69	59	52	39	35	31	20
2011-2015	229	180	142	111	96	79	47	18	14	14	12
2016-2020	229	174	127	67	31	26	9	0	0	0	0

Events

	0	12	24	36	48	60	72	84	96	108	120
2006-2010	0	16	38	46	50	56	56	56	57	58	58
2011-2015	1	44	64	73	76	80	85	86	87	87	88
2016-2020	2	38	64	74	78	78	79	79	79	79	79

Leiomyosarcoma

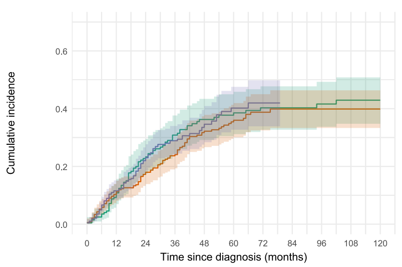

At Risk

	0	12	24	36	48	60	72	84	96	108	120
2006-2010	211	171	134	103	79	72	58	43	39	35	29
2011-2015	344	275	220	181	137	105	61	22	19	18	16
2016-2020	391	298	226	138	58	45	12	0	0	0	0

Events

	0	12	24	36	48	60	72	84	96	108	120
2006-2010	1	21	44	57	65	68	70	70	71	72	72
2011-2015	2	36	55	71	90	97	101	102	102	102	102
2016-2020	2	41	77	93	100	105	106	106	106	106	106

Undifferentiated pleomorphic sarcoma

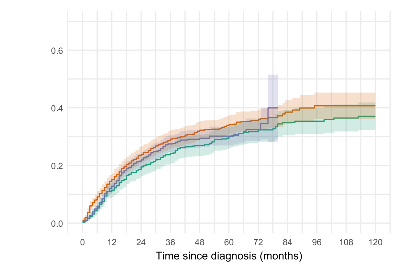

At Risk

	0	12	24	36	48	60	72	84	96	108	120
2006-2010	665	512	436	354	285	252	197	127	111	98	71
2011-2015	987	754	605	483	381	315	190	82	73	62	43
2016-2020	1130	861	662	387	192	155	41	0	0	0	0

Events

	0	12	24	36	48	60	72	84	96	108	120
2006-2010	2	70	116	140	153	165	173	179	180	182	183
2011-2015	9	140	213	253	272	282	289	294	297	297	297
2016-2020	5	133	219	260	268	270	274	276	276	276	276

Myxoid liposarcoma

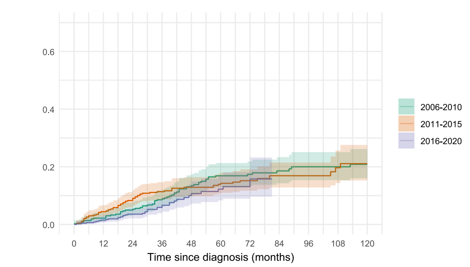

At Risk

	0	12	24	36	48	60	72	84	96	108	120
2006-2010	377	353	326	290	231	207	161	115	101	94	79
2011-2015	456	397	362	301	268	236	146	71	65	54	41
2016-2020	487	449	393	245	127	101	32	0	0	0	0

Events

	0	12	24	36	48	60	72	84	96	108	120
2006-2010	1	8	19	31	44	53	54	56	58	58	59
2011-2015	0	19	38	47	52	56	58	60	60	62	63
2016-2020	0	7	16	26	33	35	37	37	37	37	37

Figure S14. Trends in cumulative mortality by histological subtype using cumulative incidence function

Separate models were created for the major histological subtypes and the cumulative incidence was visualized. Consistent with the main analysis, only Ewing sarcoma showed a decreasing trend in cumulative incidence over time. Using the entire follow-up period allowed for a long-term prognosis estimation, making this trend more evident.
